# QTL meta-analysis provides a comprehensive view of loci controlling partial resistance to *Aphanomyces euteiches* in four sources of resistance in pea

**DOI:** 10.1186/1471-2229-13-45

**Published:** 2013-03-16

**Authors:** Céline Hamon, Clarice J Coyne, Rebecca J McGee, Angélique Lesné, Robert Esnault, Pierre Mangin, Marie Hervé, Isabelle Le Goff, Gwenaëlle Deniot, Martine Roux-Duparque, Gérard Morin, Kevin E McPhee, Régine Delourme, Alain Baranger, Marie-Laure Pilet-Nayel

**Affiliations:** 1 INRA, UMR1349 IGEPP, Le Rheu F-35653, France; 2 USDA, ARS, Western Regional Plant Introduction Station, Washington State University, Pullman, WA, 99164-6402, USA; 3 USDA, ARS, Grain Legume Genetics and Physiology Research Unit, Pullman, WA, 99164-6434, USA; 4 INRA, Domaine Expérimental d’Epoisses, UE0115, Bretenières, F-21110, France; 5 GSP, Domaine Brunehaut, Estrées-Mons, 80200, France; 6 Department 7670, North Dakota State University, 370G Loftsgard Hall, Fargo, ND, 58108-6050, USA; 7 Current address: Vegenov-BBV, Penn ar Prat, Saint Pol de Léon, 29250, France; 8 Current address: INRA, UMR1301 IBSV Interactions Biotiques en Santé Végétale, 400 route des Chappes, Sophia Antipolis Cedex, 06903, France; 9 Current address: HM CLAUSE, 1 chemin ronzières, La Bohalle, 49800, France; 10 Current address: Chambre d'Agriculture de l'Aisne, 1 rue René Blondelle, Laon Cedex, 02007, France

**Keywords:** Aphanomyces root rot, *Pisum sativum*, Meta-QTL, QTL diversity, Consistent genomic regions, Resistance alleles, Undesirable alleles

## Abstract

**Background:**

Development of durable plant genetic resistance to pathogens through strategies of QTL pyramiding and diversification requires in depth knowledge of polygenic resistance within the available germplasm. Polygenic partial resistance to Aphanomyces root rot, caused by *Aphanomyces euteiches,* one of the most damaging pathogens of pea worldwide, was previously dissected in individual mapping populations. However, there are no data available regarding the diversity of the resistance QTL across a broader collection of pea germplasm. In this study, we performed a meta-analysis of Aphanomyces root rot resistance QTL in the four main sources of resistance in pea and compared their genomic localization with genes/QTL controlling morphological or phenological traits and with putative candidate genes.

**Results:**

Meta-analysis, conducted using 244 individual QTL reported previously in three mapping populations (Puget x 90–2079, Baccara x PI180693 and Baccara x 552) and in a fourth mapping population in this study (DSP x 90–2131), resulted in the identification of 27 meta-QTL for resistance to *A. euteiches*. Confidence intervals of meta-QTL were, on average, reduced four-fold compared to mean confidence intervals of individual QTL. Eleven consistent meta-QTL, which highlight seven highly consistent genomic regions, were identified. Few meta-QTL specificities were observed among mapping populations, suggesting that sources of resistance are not independent. Seven resistance meta-QTL, including six of the highly consistent genomic regions, co-localized with six of the meta-QTL identified in this study for earliness and plant height and with three morphological genes (*Af, A, R*). Alleles contributing to the resistance were often associated with undesirable alleles for dry pea breeding. Candidate genes underlying six main meta-QTL regions were identified using colinearity between the pea and *Medicago truncatula* genomes.

**Conclusions:**

QTL meta-analysis provided an overview of the moderately low diversity of loci controlling partial resistance to *A. euteiches* in four main sources of resistance in pea. Seven highly consistent genomic regions with potential use in marker-assisted-selection were identified. Confidence intervals at several main QTL regions were reduced and co-segregation among resistance and morphological/phenological alleles was identified. Further work will be required to identify the best combinations of QTL for durably increasing partial resistance to *A. euteiches*.

## Background

Polygenic resistance frequently contributes to partial but presumably more durable levels of resistance in cultivated crops [1] and is a major target for many breeding programs. Pressure to reduce chemical applications and frequent breakdown of major resistance genes in plant species encourage the integration of polygenic resistance into cultivars of many crops. Polygenic resistance is controlled by many Quantitative Trait Loci (QTL), which express minor to major effects and whose functional roles are usually poorly understood [1,2]. The combined and diversified use of multiple major or minor-effect resistance genes should result in both increasing levels of resistance [3-6] and in limiting the adaptation of pathogen populations to plant genetic resistances [7-9]. The mining of diverse loci and gene functions controlling polygenic resistance, combined and deployed together with available major resistance genes, is expected to be a key strategy for durably improving plant genetic resistance to pathogens. A depth knowledge of the diversity of QTL controlling polygenic resistance among genetic resources of cultivated crop species is necessary for long-term genetic improvement. St-Clair [10] reviewed diversity studies of resistance QTL in several bi-parental populations from multiple germplasm sources and the subsequent use of marker-assisted-selection (MAS) for diverse resistance QTL. Linkage mapping in multi-parental population [11] and association mapping in connected recombinant inbred lines or natural populations [12,13] have been developed for more powerful detection and comparison of positive alleles among a wider diversity of genotypes than in previous studies. Whole genome sequencing data in conjunction with genome-wide association genetics has been shown a promising approach to investigate the diversity of disease resistance QTL in plants and to detect with high resolution common resistance alleles in a given population [2,14]. QTL meta-analysis [15] have also been shown a useful approach to obtain a synthetic representation of the diversity of loci controlling a trait of interest in plants. It consists of combining results from previous QTL reports into consensus genomic regions, or meta-QTL, associated with the trait variation. The position of these meta-QTL is estimated with higher accuracy compared to position estimates of individual QTL. Statistical methods for meta-analysis of QTL have been implemented in two main software packages, Biomercator [16] and Meta-QTL [17]. This approach has recently been applied in plants [18-21] and is especially useful for obtaining an overview of loci and for increasing resolution to identify candidate genes controlling polygenic resistance [22-29].

Aphanomyces root rot, caused by the soilborne oomycete *Aphanomyces euteiches* Drech., is one of the most damaging pea diseases worldwide [30] and is a major limiting factor to pea production in France. The absence of adequate chemical or cultural methods to manage the disease requires development of pea cultivars with acceptable levels of resistance to sustain pea production. In pea, resistance to *A. euteiches* has been shown to be partial, polygenically inherited [31] and correlated with undesirable traits such as long internods, anthocyanin production and late-flowering [32] (Roux-Duparque, Pers. Comm.). Previous exploration studies of diversity within *Pisum* for resistance to *A. euteiches* reported that sources of resistance were scarce [33-35] (Pilet-Nayel *et al.* unpublished data). The few sources of resistance identified, were integrated into pea breeding programs for root rot resistance over the past 30 years. These programs resulted in the release of germplasm with acceptable agronomic characteristics and increased levels of partial resistance in the USA [36-42] and in France [43]. In the past 10 years, a collaborative French-American research program conducted genetic dissection of polygenic resistance to *A. euteiches* in four main sources of resistance (90–2079, 90–2131, PI180693 and 552) derived from and/or integrated into various breeding programs. These sources of resistance showed the highest and/or most stable levels of resistance across several different field locations in France and in the USA. They presented different genealogies [44], except PI180693 and 90–2131 which were related [38] but differed in their levels of resistance. Results from this collaborative program have been reported for three mapping populations [31,45,46]. From a RIL population derived from the cross Puget (susceptible to *A. euteiches*) × 90–2079 (resistant to *A. euteiches* in the USA), 14 QTL were associated with Aphanomyces root rot resistance, including three QTL (*Aph1*, *Aph2* and *Aph3*) that were detected over various environments and isolates [31,45]. In two RIL populations derived from crosses between the susceptible variety Baccara and the resistant germplasm lines PI180693 and 552, a total of 23 genomic regions controlling partial resistance to *A. euteiches* were identified. These regions included five highly stable regions across environments (France and USA), isolates and genetic background, as well as 13 epistatic interactions [46]. QTL results obtained from a fourth RIL population derived from the cross between DSP (susceptible) and 90–2131 (resistant) are reported in this manuscript along with QTL comparisons between the independent mapping studies. This comparison will provide a more complete view of the diversity of Aphanomyces resistance loci available from the four sources of resistance. Greater understanding of the resistance QTL will allow QTL pyramiding and diversification strategies to be developed, in an effort to prevent the pathogen from overcoming the crucial and limited level of resistance. Comparisons between Aphanomyces resistance QTL and QTL controlling other agronomic traits will aid selection for positive associations and avoid selection of negative linkages between resistance and undesirable alleles.

The objectives of this study are (i) to provide an overview of the diversity and the structural genomic organization of QTL involved in resistance to *A. euteiches* in pea, by compiling QTL mapping data obtained from four main sources of resistance, (ii) to identify positive and negative co-segregations between Aphanomyces resistance alleles and alleles controlling morphological and phenological traits and (iii) to identify putative candidate genes underlying reduced confidence intervals of main meta-QTL using pea-*M. truncatula* translational genomics. We conducted a meta-analysis of QTL for resistance to *A. euteiches* in pea integrating (i) QTL previously detected for resistance to *A. euteiches* from three resistance sources (90–2079, 552, PI180693) [31,45,46] and (ii) QTL reported for the first time in this study from the additional source of resistance, 90–2131. We also performed a meta-analysis of QTL controlling morphological and phenological traits (plant height and earliness), from phenotypic data and QTL results obtained in three of the four RIL populations studied for Aphanomyces resistance. Finally, we compared genomic localizations of the identified morphological and phenological meta-QTL to the ones of Aphanomyces resistance meta-QTL. We identified candidate genes underlying main resistance meta-QTL using the pea-*M. truncatula* translational toolkit of Bordat *et al.*[47].

## Results

### Genetic analysis of resistance to *A. euteiches* in the DSP × 90–2131 RIL population

**Figure 1:**
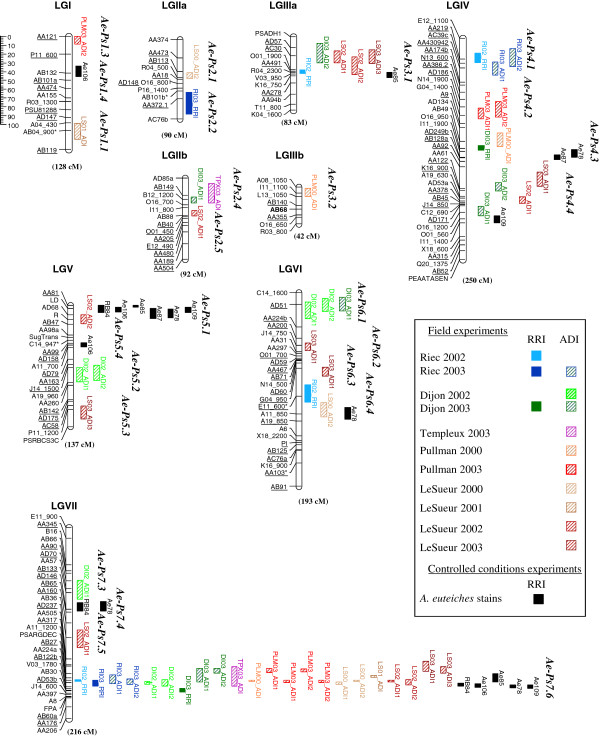
**Genomic localization of QTL for resistance to *A. euteiches *in the DSP x 90–2131 RIL population. **Genetic map constructed from 111 RILs derived from the cross DSP × 90–2131 and genomic localization of additive-effect QTL detected for Aphanomyces root rot resistance in 11 field environments over four years and five locations and against six strains (RB84, Ae106, Ae85, Ae87, Ae78 and Ae109) in controlled conditions, using two resistance criteria (Root Rot and Aerial Decline Indexes, RRI and ADI, respectively). Linkage groups (LG) assigned to the SSR pea reference map (Loridon *et al., *2005) are named from I to VII. The size of each LG is indicated in cM Kosambi. Marker names are indicated on the left of each LG. Markers with biased allelic segregation are indicated by one asterisk. Markers common to the SSR pea reference map (Loridon *et al., *2005) are underlined. Lengths of additive-effect QTL boxes correspond to the LOD-1 support interval from the peak marker.

The genetic map, constructed from the DSP × 90–2131 RIL population, was comprised of a total of 168 markers, including 107 SSRs, 56 RAPDs, three genes of known-function (two flowering related genes, LD and FPA [48], and a putative sugar transporter, SugTrans [49]) and two morphological markers (*r* and *Pl*) distributed over nine linkage groups (LG) (Figure 1). The map covered 1046 cM Kosambi, corresponding to about 77% of the pea SSR reference genetic map [50]. Five of the 168 markers (3%) showed significant distorsion from the expected Mendelian ratio (α=0.01). Of the 168 markers, 86 (51%), equally distributed over nine LG, were common to the pea reference genetic map [50], including 74 SSR, 11 RAPD and one morphological marker (*Pl*). These common markers were ordered with high colinearity between the two genetic maps, except in the distal part of LGIIb, where the order of a block of markers was inverted. Compared to the reference map, the distal part of LGI and the pericentromeric regions of LGII and LGIII remain uncovered by markers on the DSP × 90–2131 map.

Analysis of the DSP × 90–2131 RIL resistance data in controlled conditions showed that the five pea lines used as controls ranked as expected for root rot index (RRI) scores with all but one of the six *A. euteiches* strains, confirming the strain pathotypes as described in [51] and [52] (Additional file 1). RRI scores in the DSP × 90–2131 RILs showed highly significant genotypic and block effects (*P* < 0.001) for each strain with the exception of Ae85. Mean-based heritability of RRI was moderate (*h*^2^ = 0.55 with Ae85 strain) to high (*h*^2^ = 0.91 with RB84 strain). Distribution of RRI adjusted means in the RIL population tended to fit a normal curve with each of the six strains except RB84 (Additional file 2). Lower levels of symptoms were observed in the RIL population with the Ae85 strain than with the five other strains (μ = 1.9 and 2.9-3.6, respectively). Transgressive RIL segregants with increased resistance and susceptibility compared to parental values were observed with each of the six strains.

Analysis of the DSP x 90–2131 RIL resistance data in each of the 11 field environments studied showed highly significant genotypic effect (*P*≤0.002) for all but two variables (RI-2002-RRI, RI-2003-ADI1) and significant block effects (*P*≤0.002) for half of the variables. Mean-based heritabilities of traits, ranging from 0.25 (RI-2003-ADI1) to 0.87 (PLM-2000-ADI1), were low (*h*^2^ ≤ 0.40) for five variables, moderate (0.40 < *h*^2^ ≤ 0.70) for nine variables and high (h^2^ > 0.70) for six aerial decline index (ADI) variables (Additional file 3). Frequency distribution of the adjusted mean scores for each variable in the RIL population tended to fit normal curves, with the exception of the ADI scores obtained in Pullman in 2000 and 2003 (Additional file 3). Differences were observed between means and ranges of the RIL population for RRI and ADI variables, depending on the location and year. In all experiments, transgressive segregants, either more susceptible or more resistant than the parents, were observed.

Half of the DSP × 90–2131 RIL phenotypic data from different field criteria and environments were significantly (*P* < 0.001) and positively correlated with most of the other field data (Additional file 4). Data with low heritability values were mostly poorly correlated with the other field data (RI-2002-RRI, RI-2003-ADI1, DI-2003-RRI and LS-2003-ADI1). Data from evaluations in controlled conditions for the six *A. euteiches* strains were highly correlated (*P* < 0.001) with each other. The RB84 strain data was also positively and significantly correlated with most of the RRI and ADI scores assessed in French and American field conditions, contrary to the other strain data which were significantly correlated with few or no field scoring data.

A total of 79 additive-effect QTL, corresponding to 25 genomic regions distributed over nine linkage groups, were detected in the DSP × 90–2131 RIL population for Aphanomyces root rot resistance evaluated in 11 field environments and with six strains in controlled conditions (Figure 1, Additional file 5). A major QTL, *AePs7.6*, previously identified in [46], was consistently detected on LGVII from ADI and/or RRI data collected in the 11 field environments studied and for five of the six strains in controlled conditions. The *Ae-Ps7.6* genomic region covered about 35 cM and accounted for the majority of the phenotypic variation, yielding 59.8% for resistance to the RB84 strain. Two QTL, *Ae-Ps3.1* and *Ae-Ps5.1*, previously identified [46], were also consistently detected on LGIIIa and LGV (near to the *R* locus), especially from ADI and/or RRI field data (*Ae-Ps3.1*) and from the six strain data in controlled conditions (*Ae-Ps5.1)*. Five genomic regions on LGIV and LGVI (*Ae-Ps4.1, Ae-Ps6.1*, *Ae-Ps4.3, Ae-Ps4.4* and *Ae-Ps6.4)*, were consistently associated with three to five individual field and/or controlled condition resistance QTL. Seventeen QTL, distributed on the seven LG, were less consistently identified from one or two variable(s) of the same criterion. For the eight total genomic regions detected from at least three variables, no consistent specificities were observed across either environments or strains, except for the three regions that were more specifically identified from French field (*Ae-Ps4.1* and *Ae-Ps6.1*) or controlled condition (*Ae-Ps5.1*) data. Four of the eight regions (*Ae-Ps7.6, Ae-Ps5.1, Ae-Ps4.4* and *Ae-Ps4.3*) explained more than 15% of the phenotypic variation observed for at least one variable. Resistance was contributed by 90–2131 and DSP alleles, whatever the variable, at four (*Ae-Ps7.6, Ae-Ps5.1, Ae-Ps3.1, Ae-Ps4.1*) and two (*Ae-Ps4.4, Ae-Ps6.1*) of the eight regions, respectively. Four significant pairwise epistatic interactions were identified for increasing resistance to *A. euteiches*, especially to the RB84 strain. All the interactions involved at least one minor QTL, especially *Ae-Ps7.3* (Additional file 6).

### Genetic analysis of earliness and plant height from DSP × 90–2131, Baccara × PI180693 and Baccara x 552 RIL populations

Analysis of variance of earliness and plant height data in each environment and population showed a significant genotypic effect (*P* < 0.0001) and no significant block effect (*P* > 0.05) for all variables. Mean-based heritabilities of the flowering traits (FLO) and plant height variables (HT) evaluated in a three-block design were very high (*h*^2^ > 0.80), except for FLO1 assessed at Dijon in 2003 (Additional file 7). Frequency distributions of the FLO and HT scores closely followed normal curves in the DSP × 90–2131 and Baccara × 552 populations. FLO distributions tended to fit bimodal curves in the Baccara × PI180693 population in most of the environments tested, despite the similar values of parental lines. The FLO and HT scores were significantly (*P* < 0.001) and negatively correlated with no, few and most Aphanomyces resistance variable scorings in the Baccara × 552, DSP × 90–2131 and Baccara × PI180693 populations, respectively (data not shown). FLO and HT values were highly correlated in each population, across all environments (infested/healthy).

**Figure 2:**
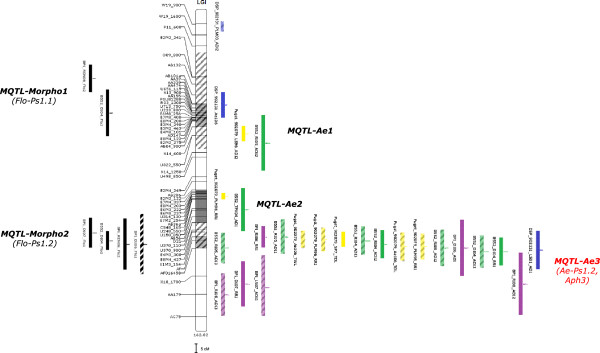
**Localization of individual QTL and meta-QTL for resistance and morphological traits onto the consensus marker map: linkage group I. **Individual QTL and meta-QTL detected are represented on the right of each linkage group (LG) for resistance to *A. euteiches *and on the left of each LG for morphological and phenological traits. Marker names are indicated on the left of each LG of the consensus map. Genetic distances between markers are represented in cM Kosambi, according to Additional file 10. Each LG size is indicated in cM Kosambi at the bottom of the LG. LOD-1 confidence interval of initial QTL detected for resistance to *A. euteiches *from Puget x 90–2079, DSP x 90–2131, Baccara x PI180693 and Baccara x 552 RIL populations are indicated by yellow, blue, purple and green boxes, respectively. LOD-1 confidence interval of initial QTL detected for earliness and plant height are indicated by black bars. Names of initial QTL are coded according to RIL population, field environment and year, *A. euteiches *isolate and scoring criterion, as mentioned in Additional file 5 of this study and in Hamon *et al.*[46]. Redundant initial QTL which were not considered in the meta-analysis are indicated in hatched boxes. Main genomic regions comprising overlapping individual QTL are named “*Ae-Ps*” or “*Aph*” for resistance to *A. euteiches *according to [46] and [45], respectively, and “*Flo-Ps*” or “*HT-Ps*” for earliness and plant height, respectively. Meta-QTL are named “*MQTL-Ae*” and “*MQTL-Morpho*” for resistance to *A. euteiches* and morphological/phenological traits (earliness, plant height), respectively. Meta-QTLs highlighted in red correspond to the 7 main consistent genomic regions identified for resistance to *A. euteiches *(Table 3). Confidence intervals of meta-QTL are represented by grey and hatched boxes in the width of each LG for resistance to *A. euteiches *and morphological/phenological traits, respectively.

A total of 33 additive-effect QTL corresponding to 14 genomic regions were detected for the flowering traits (FLO1, FLO2), from the three RIL populations studied. Additionally, three additive-effect QTL corresponding to three genomic regions were detected for plant height (HT) (Additional files 8 and 9, and Figure 2). No co-localization was observed between genomic regions detected for FLO and HT traits. Four genomic regions were consistently detected for FLO, each containing four to six individual QTL. Three of them (*Flo-Ps2.2, Flo-Ps3.1, Flo-Ps7.2)* explained up to 40.8% of the phenotypic variation while *Flo-Ps1.2* explained up to 10% of the variation. Two other regions (*Flo-Ps1.1* and *Flo-Ps6.3*) were also identified, each containing two individual QTL. *Flo-Ps6.3* explained up to 40% of the phenotypic variation. Among the six regions, alleles for earliness at *Flo-Ps1.1, Flo-Ps2.2* and *Flo-Ps3.1* were contributed by Baccara, while they were contributed by 90–2131 at *Flo-Ps6.3* and PI180693 or 552 at *Flo-Ps1.2* and *Flo-Ps7.2*. Eight FLO QTL with minor effects (*R*^2^ = 3.5 to 16.3%), were specifically detected in one environment. The three QTL identified for plant height were detected with minor-effects, and at these loci, the 90–2131 alleles contributed to shorter plants.

### QTL meta-analysis and co-localisations between Aphanomyces resistance and morphological/phenological meta-QTL

QTL meta-analysis of Aphanomyces resistance and morphological/phenological traits was performed using three mapping studies in the four pea RIL populations described in Table 1. The QTL mapping study conducted using the Puget × 90–2079 RIL population [31,45] was updated by adding 53 markers (41 SSRs and 12 RAPDs) to the genetic map. Thirty five of the added markers were common to at least one of the two other individual genetic maps used. The updated Puget × 90–2079 genetic map, covering 1523 cM, comprised a total of 377 markers (data not shown). Individual QTL detected on the previous map [31,45] were confirmed and the updated QTL information were used for the meta-analysis.

**Table 1:** QTL mapping populations and studies used for meta-analysis

**Cross**	**Populations size, type and generation**	**Donor**	**Genetic map**	**Phenotypic evaluation**	**QTL detection method**^**a**^	**Reference**
Puget x 90-2079	127 RIL (F10)	USDA-ARS (USA)	324 markers	Resistance to *A. euteiches*	CIM	Pilet-Nayel *et al.*[31,45]
				Resistance to *A. euteiches*		
Baccara x PI180693	178 RIL (F8)	INRA (France)	224 markers (consensus genetic map)	Morphological and phenological traits	CIM	Hamon *et al.*[46]
				Resistance to *A. euteiches*		
Baccara x 552	178 RIL (F9)	INRA (France)		Morphological and phenological traits Resistance to *A. euteiches*	CIM	Hamon *et al.*[46]
				Resistance to *A. euteiches *resistance		
DSP x 90-2131	111 RIL (F10)	USDA-ARS (USA)	168 markers	Morphological and phenological traits	CIM	This study

^a ^Composite interval mapping.

#### Consensus map

The consensus marker map was established from the three individual genetic maps (Puget × 90–2079, DSP × 90–2131, Baccara × PI180693/552) using the Meta-QTL software. The map comprised a total of 619 markers (31% SSR; 33% RAPD; 32% AFLP; 2.4% STS, 1.6% isozyme or morphological markers) and covered 1513 cM Kosambi (Additional file 10). Eighty-six percent of the markers mapped to only one of the individual maps studied, while 14% mapped to two or three individual maps with consistent marker order and regular distribution on all LG (8 to 22 common markers per LG) with exception of LGI. Fifteen percent of the 619 markers of the consensus map were also mapped on the reference genetic map described by [53] and had consistent positions between the two maps.

#### Meta-QTL for resistance to A. euteiches

A total of 244 individual additive-effect QTL detected for resistance to *A. euteiches* in the three mapping studies were projected onto the consensus marker map (Figure 2). Projection of all individual QTL allowed three to five main genomic regions per LG to be identified, each consisting of individual QTL with overlapping confidence intervals. Removal of redundant or single-individual QTL left 115 independent QTL for the meta-analysis. Meta-analysis identified 27 meta-QTL for resistance to *A. euteiches* distributed over the seven LG, with three to four meta-QTL per LG (Table 2, Figure 2). Each meta-QTL was comprised of 1.2 to 11.8 independent QTL (mean = 4.2 ± 2.5 independent QTL per meta-QTL) corresponding to one to 41 initial individual QTL (mean = 9 ± 8 initial QTL per meta-QTL). Confidence intervals of meta-QTL ranged from 0.9 to 53.1 cM (Kosambi), with a mean of 13.7 ± 12 cM, and were lower than 20 cM for 85% of the meta-QTL. The average width of the independent QTL confidence intervals was reduced from 0.9-fold to 28.4-fold (4.1 ± 5.2 in average), depending on the meta-QTL. Among the 27 meta-QTL identified, 11 were highly consistent, including eight meta-QTL comprised of at least 12 initial individual QTL and three meta-QTL comprised of eight to 11 initial QTL (Table 2). The remaining 16 less consistent meta-QTL aggregated individually between one and six initial QTL. The 11 consistent meta-QTL corresponded to ten independent genomic regions. In seven of these regions, initial QTL were clustered in highly consistent positions, namely on LGI (*MQTL-Ae3*), LGII (*MQTL-Ae5*), LGIII (*MQTL-Ae8/Ae9*), LGIV (*MQTL-Ae12* and *MQTL-Ae15*), LGV (*MQTL-Ae16/Ae17*) and LGVII (*MQTL-Ae25/Ae26*). Eight of the 11 consistent meta-QTL aggregated initial QTL detected from either three or four mapping populations, and all but one of the remaining meta-QTL were detected from two mapping populations. At each meta-QTL that contained initial QTL from at least two mapping populations, two to five parental alleles, mostly from the resistant parent, contributed to resistance with a mean of 3.2 alleles per meta-QTL. Five of the 24 meta-QTL comprised of initial QTL from at least two field environments were specific to either French (*MQTL-Ae12, MQTL-Ae20/Ae21*) or USA (*MQTL-Ae13, MQTL-Ae17*) environments, while the remaining 19 were not environment-specific. All meta-QTL including initial QTL detected from controlled condition data co-located with initial QTL identified from at least one field environment. None of the meta-QTL comprised of a minimum of two initial controlled condition QTL, were specific to a single strain. Eighty percent of the meta-QTL were not specific to root or aerial scoring criteria used in the different studies, even if some of them included initial QTL that were primarily detected from root criteria (*MQTL-Ae16*) or aerial criteria (*MQTL-Ae27*).

**Table 2:** **Meta-QTL detected for resistance to *A. euteiches *and morphological/phenological traits**

**LG**	**Meta-QTL name**^**a**^	**Position onto the consensus map (cM)**^**b**^	**Closest left marker from the position**	**CI of Meta- QTL (cM)**^**c**^	**Number of independent QTL per meta-QTL**	**Mean ± Standard deviation of independant QTL CI (cM)**^**d**^	**Number of initial QTL per Meta- QTL**^**e**^	***R***^***2 ***^**range of initial QTL (%)**^**f**^	**CI range of initial QTL (cM)**^**g**^	**Trait-improving alleles at initial QTL**^**h**^							**Initial QTL name (Pilet-Nayel *et al.***[31,45]**; Hamon *et al.***[46]**; this study)**
										552	PI180693	90- 2131	90- 2079	Baccara	DSP	Puget	
Resistance to *A. euteiches*																	
l	*MQTL-Ae1* *	56.2	E2M2_275	**19.4**	**3**	35.9 ± 10.0	**3**	6.0-7.9	7.0-26.0	**1**		**0**	**1**	**0**	**1**	**0**	*Ae-Ps1.1*
	*MQTL-Ae2*	99.5	U248_550	22.7	1.9	31.4 ± 1.1	**2**	4.0-16.9	3.0-26.0	**0**			**1**	**1**		**0**	*Ae-Ps1.2*
	***MQTL-Ae3*** **	**126.1**	**AF016458**	**3.8**	**8.1**	**24.5 ± 8.3**	**19**	**4.0-12.0**	**20.0-30.0**	**7**	**5**	**1**	**3**	**1**	**0**	**2**	***Aph3/Ae-Ps1.2***
ll	*MQTL-Ae4*	14.9	G10_700	13.0	**2**	22.9 ± 12.1	**6**	9.0-15.0	7.0-22.0	**4**		**0**		**1**	**1**		*Ae-Ps2.1*
	***MQTL-Ae5***	**69.3**	A	4.8	**6.8**	**28.2 ± 23.4**	**12**	**6.0-23.0**	**3.0-25.0**	**3**	**7**	**0**	**1**	**0**	**1**	**0**	***Ae-Ps2.2***
	MQTL-Ae6 *	**91.5**	**A19_800**	15.3	**1.2**	**15.5 ± 0.0**	**1**	**27.0**	**13.0**		**1**		**0**				***Ae-Ps2.2***
	*MQTL-Ae7*	**192.4**	**AA205**	7.6	**3**	38.3 ± 28.2	**6**	5.0-13.4	**3.0-22.0**		**0**	**1**	**1**	**2**	**2**	**0**	*Ae-Ps2.3*
lll	***MQTL-Ae8*** *	**40.9**	**X03_1000**	6.9	**3.9**	**13.6 ± 6.2**	**18**	**7.0-30.0**	**3.0-15.0**	**6**	**12**			**0**			***Ae-Ps3.1***
	***MQTL-Ae9***	**58.4**	**AB70**	11	**4.4**	**17.3 ± 4.4**	**5**	**6.0-14.0**	**4.0-20.0**	**4**	**1**			**0**			***Ae-Ps3.1***
	*MQTL-Ae10*	**127.8**	PSU81287	44.6	**3.1**	79.1 ± 7.6	**6**	6.0-9.1	4.0-23.0			**6**			**0**		*Ae-Ps3.1*
	*MQTL-Ae11*	170.5	AB122a	16.2	3.5	45.9 ± 38.0	**5**	4.0-9.0	5.0-15.0		**1**	**1**		**3**	**0**		*Ae-Ps3.2*
IV	***MQTL-Ae12***	**26.8**	**AA430942**	**5.8**	**7.4**	**39.0 ± 44.7**	**17**	**3.0-25.0**	**2.0-30.0**	**7**	**3**	**3**	**0**	**3**	**0**	**1**	***Ae-Ps4.1***
	*MQTL-Ae13*	78.7	E3M6_431	17.7	2.3	28.1 ± 11.1	4	3.0-17.0	2.0-17.0	0		0	1	1	2	0	*Ae-Ps4.2*
	*MQTL-Ae14*	133.6	AD249b	7.5	7.5	29.1 ± 17.6	11	5.0-37.0	5.0-35.0		3	3	3	0	2	0	*Ae-Ps4.3*
	***MQTL-Ae15*** *	**172.3**	**J14_850**	**0.9**	**7.8**	**25.6 ± 26.7**	**14**	**5.0-44.0**	**3.0-18.0**	**1**	**1**	**0**	**8**	**0**	**4**	**0**	***Aph1/Ae-Ps4.4/Ae-Ps4.5***
V	***MQTL-Ae16***	**6.2**	**LD**	**11.8**	**2.5**	**17.9 ± 4.3**	**8**	**7.0-37.8**	**2.0-23.0**	**1**		**6**		**1**	**0**		***Ae-Ps5.1***
	***MQTL-Ae17***	**22.6**	**E8M2_280**	**9.1**	**4.6**	**32.0 ± 23.9**	**6**	**7.2-23.9**	**2.0-16.0**	**0**		**1**	**4**	**1**	**0**	**0**	***Aph2/Ae-Ps5.1***
	*MQTL-Ae18*	105.9	U352_120	15.0	3.9	42.6 ± 31.8	5	4.4-17.5	5.0-20.0	1		2	2	0	0	0	*Ae-Ps5.2*
	*MQTL-Ae19*	132.9	A04_400	53.2	2	46.2 ± 19.8	2	6.0-11.8	15.0.27.0	1		1		0	0		*Ae-Ps5.3*
VI	MQTL-Ae20 *	2.4	E09_1400	10.1	3.1	24.2 ± 13.2	3	11.0-49.0	10.0-32.0	0	1			2			*Ae-Ps6.1*
	*MQTL-Ae21*	39.7	K16_400	28.7	1.9	42.3 ± 11.6	4	6.9-14.0	6.0-18.0			0	0		3	1	*Ae-Ps6.1*
	*MQTL-Ae22*	71.9	AA200	10.7	2	22.4 ± 15.7	4	7.0-13.7	8.0-13.0	3		1		0	0		*Ae-Ps6.2/Ae-Ps6.3*
	*MQTL-Ae23*	121.3	PSU31669	10.9	4	30.9 ± 13.7	4	5.0-14.8	13.0-19.0			2	0		1	1	*Ae-Ps6.4*
VII	*MQTL-Ae24*	91.8	E8M3_446	10.8	3.8	55.5 ± 45.2	10	5.0-12.0	6.0-28.0	1		1		6	2		*Ae-Ps7.2/Ae-Ps7.3/Ae-Ps7.4*
	***MQTL-Ae25***	**155.3**	**IJB174**	**6.6**	**5.3**	**17.6 ± 5.6**	**13**	**6.0-38.0**	**2.0-22.0**	**1**	**3**	**8**	**0**	**0**	**0**	**1**	***Ae-Ps7.6a***
	***MQTL-Ae26***	**184.4**	**AB122b**	**3.8**	**11.8**	**19.8 ± 20.3**	**41**	**6.0-60**	**1.0-21.0**	**9**	**12**	**20**		**0**	**0**		***Ae-Ps7.6a***
	*MQTL-Ae27* *	205.5	AA176	1.3	3.4	9.5 ± 4.8	13	5.0-42.0	4.0-12.0		1		1	11		0	*Ae-Ps7.6b*
Morphological/Phenological traits																	
I	*MQTL-Morpho1* *	43.7	AB101a	28.0	2	42.5 ± 13.0	2	3.0-5.0	13.0-22.0	0	0			2			*Flo-Ps1.1*
	*MQTL-Morpho2* **	120.9	*Af*	12.9	3	44.1 ± 31.9	4	2.0-10.0	9.0-28.0	1	3			0			*Flo-Ps1.2*
II	*MQTL-Morpho3* *	82.2	AB50	0.84	2	31.2 ± 39.0	6	10.0-41.0	0.5-8.0		0	1		5	0		*Flo-Ps2.1/Flo-Ps2.2*
III	*MQTL-Morpho4* *	41.7	A04_450	1.2	2	6.1 ± 0.2	6	14.0-33.0	5.0-8.6		0			6			*Flo-Ps3.1*
IV	*MQTL-Morpho5* *	175.3	AD171	16.8	3	37.8 ± 21.1	3	8.0-11.0	10.0-50.9	1		1		0	1		*Flo-Ps4.1/Flo-Ps4.2/Ht-Ps4.1*
VI	*MQTL-Morpho6*	87.6	AA297	6.0	1	6.0 ± 0.0	3	13.9-40.0	2.0-6.0	0		2		1	0		*Flo-Ps6.2/Flo-Ps6.3*
VII	*MQTL-Morpho7* *	205.2	AA176	0.67	2	4.9 ± 1.5	6	27.0-41.0	3.0-7.8		6			0			*Flo-Ps7.2*

^a ^Meta-QTL names are coded as follows : *MQTL-Ae* = Meta-QTL detected for resistance to *A. euteiches*; *MQTL-Morpho* = Meta-QTL detected for morphological and phenological traits (flowering time and plant height). On each Linkage Group (LG), *MQTL-Ae *and *MQTL-Morpho, *which co-localize, are mentioned by asterisks. Meta-QTL indicated in bold correspond to the seven main consistent genomic regions identified for resistance to *A. euteiches*.

^b ^QTL position at the LOD (Log of likelihood ratio) score peak, from the first marker of the linkage group in cM Kosambi.

^c ^95% confidence interval of the meta-QTL given by MetaQTL software, in cM Kosambi.

^d ^Mean and standard deviation of confidence intervals (estimates according to [55]) of all independent QTL belonging to each meta-QTL, in cM Kosambi.

^e ^Number of initial QTL (redundant and independent) underlying each meta-QTL.

^f ^Range of percentage of phenotypic variance (*R*^*2*^) explained by individual initial QTL underlying each meta-QTL.

^g ^Range of LOD-1 confidence intervals of all initial QTL underlying each meta-QTL.

^h ^Number of variables for which each RIL parental allele contributed to increased resistance to *A. euteiches*, increased earliness or short plants, at each meta-QTL. No values indicate missing data.

#### Meta-QTL for morphological/phenological traits and co-localizations with Aphanomyces resistance meta-QTL

Projection of 36 initial additive-effect QTL detected for earliness and plant height onto the consensus map outlined 15 distinct genomic regions with QTL clusters, including 21 redundant or single initial QTL. Meta-analysis of the remaining 15 non-redundant initial QTL resulted in the identification of seven meta-QTL distributed over six LG (Table 2, Figure 2). Each meta-QTL was comprised of one to three independent QTL (mean = 2.0 ±0.7 independent QTL per meta-QTL), corresponding to two to six initial individual QTL. Confidence intervals of the meta-QTL ranged from 0.7 to 28 cM and were reduced by 1 to 37.1 times compared to mean confidence intervals of clustered initial QTL. The seven meta-QTL corresponded to the six most consistent FLO genomic regions (*Flo-Ps1.1, FloPs1.2, Flo-Ps2.1/2.2, Flo-Ps3.1, Flo-Ps6.2/6.3, Flo-Ps7.2*), and to a cluster of two FLO and one HT overlapping QTL (*MQTL-Morpho5*). Five of the seven meta-QTL were identified from at least two populations and from diverse en-vironments.

Based on the 33 meta-QTL that contained more than one initial QTL for either Aphanomyces resistance or for plant earliness and height, six genomic regions were detected with overlapping intervals of resistance and phenological QTL (Figure 2, Table 2). In the regions of meta-QTL co-localization, nine negative and five positive associations were found between parental alleles contributing to resistance and early flowering and/or short stature. Positive associations involved 552, PI180693 and DSP alleles. PI180693 and/or 552 alleles contributing to resistance and normal leaf type or anthocyanin production were in coupling phase in meta-QTL regions on LGI (*MQTL-Ae3*) and LGII (*MQTL-Ae5/Ae6*). 90–2079 and 90–2131 alleles contributing to resistance and round seed type were also in coupling phase in the meta-QTL region on LGV (*MQTL-Ae16/Ae17*).

### Identification of candidate genes underlying meta-QTL

Using the Pea-*Medicago truncatula* translational toolkit of Bordat *et al.*[47], a list of genes among 5460 pea Unigenes was identified as positional candidates within the support interval of all but one (*MQTL-Ae15*) of the seven highly consistent resistance meta-QTL regions. Adjacent markers to the meta-QTL, which were common between the consensus marker map developed in this study and the pea functional map of [47], were used to define intervals containing candidate genes established in [47]. A total of 318 genes underlying main meta-QTL were identified, including 14 to 91 genes per meta-QTL (Additional file 11). Out of them, 264 were similar to genes with a putative function, among which 17% had a known-function related to plant disease resistance. These genes corresponded to resistance gene analogs (RGA) or genes involved in plant-pathogen recognition (LRR, Leucine zipper proteins), genes involved in defense or signal transduction (protein kinases, enzymes of oxidative stress and cellulose synthesis, pathogenesis-related-proteins, transcription factors, heat-shock proteins, cell division proteins, cyclin-like F-Box proteins) and genes involved in resistance to other diseases. Particularly, in meta-QTL regions with smallest confidence intervals, genes coding for LRR proteins (*MQTL-Ae3*) and protein kinases (*MQTL-Ae8/Ae9*, *MQTL-Ae12*) could be suggested as good candidates. In the other meta-QTL regions, clusters of genes coding for heat shock proteins (*MQTL-Ae16/Ae17*) and a plant disease response protein-encoding gene (*MQTL-Ae26*) were also striking putative candidates to the resistance.

## Discussion

### A moderately low diversity of loci controls quantitative partial resistance to *A. euteiches* in four main sources of resistance in pea

This study is the first that provides an overview and comparison from multiple studies of genetic loci controlling resistance to *A. euteiches* in pea. It is one of the first reports developing a comprehensive picture of the genetic architecture of disease resistance in cultivated legumes [24,54]. Similar studies have proven valuable in other plant species for determining key resistance loci useful in MAS and identifying candidate genes underlying resistance QTL [22,23,26].

In this study, 244 individual QTL detected for Aphanomyces resistance from eight variables collected in four pea RIL populations over a total of 29 field environments (two–five USA-FR locations over 10 years) and 12 controlled condition assays (two–six strains per population) were analyzed for meta-analysis. A total of 27 meta-QTL were identified, including 11 consistent meta-QTL each containing more than eight initial individual QTL. Seven main genomic regions clustering numerous initial QTL with high position consistency were highlighted. The number of consistent meta-QTL identified is quite low given the four diverse partially resistant pea lines studied and the complex inheritance of partial resistance to *A. euteiches*.

The meta-QTL analysis conducted in this study accurately compared genomic positions of individual QTL identified from different studies and refined the confidence intervals of the main genomic regions associated with resistance. The consensus map preserved marker order of individual maps based on 14% of common markers, and of the reference genetic map [53] based on 15% of common markers. Identification of over-reduced meta-QTL confidence intervals was minimized by clustering individual QTL using non-redundant independent QTL with confidence intervals estimated from their *R*^2^ values [55]. This approach resulted in the identification of three to four meta-QTL per LG, and their confidence intervals were reduced four-fold, on average, compared to mean confidence intervals of the initial independent QTL. Confidence interval reduction was correlated to the number of clustered independent QTL available for refining each meta-QTL (*r*^2^ = 0.21), as shown by the smallest confidence intervals (0.9-6.9 cM) observed for the eight most consistent meta-QTL.

In contrast to previous similar meta-QTL studies of quantitative resistance [25,26], few meta-QTL specificities were observed associated with the parental source of resistance. All but one meta-QTL clustering at least two of the initial QTL were detected in two, three or four mapping populations. Each of these meta-QTL comprised of two to five alleles that contributed to the resistance. At a given meta-QTL, meta-analysis did not provide effect estimates of the different alleles, *i.e.* classification of alleles according to their effect levels. However, at several meta-QTL, variations in additive effects and *R*^2^ were observed between initial QTL detected from different RIL populations. Initial QTL detected from the DSP × 90–2131 population at *MQTL-Ae26* had higher average additive effects and *R*^2^ values (a = 0.3, *R*^2^ = 20%) than those detected from the Baccara x PI180693 (a = 0.21, *R*^2^ = 10%) and Baccara × 552 (a = 0.14, R^2^ = 10%) populations. This suggests that some parental alleles contributing to the resistance, generally detected with high consistency, could have stronger effects than others. Multiple parental alleles associated with resistance at one meta-QTL could correspond to identical or different alleles of a single gene or to clusters of closely linked genes. Despite the reduced confidence intervals obtained for several meta-QTL, fine mapping of meta-QTL will be required to determine the correct allele/gene hypothesis. According to pedigree information available in the literature, the four resistant germplasm studied are not independent and may share common resistance genes, which is supported by the low specificity of most of the meta-QTL identified in the RIL populations. PI180693 was one of the parents used in crosses to develop 90–2131 but was not reported in the genealogy of 90–2079, which derived its resistance from MN313 [36,38]. Little information is available about the parentage of 552, which was derived from several recurrent selection cycles conducted by Lewis and Gritton [56]. The literature contains no data about genetic distances between the sources of resistance to *A. euteiches* used. However, in our study, 20 meta-QTL underlied different combinations of resistance alleles from two of the four resistant parent, frequently including 552 alleles, and nine meta-QTL included resistance alleles derived from three of the four sources of resistance. These results suggest that a common genetic background was used in the different Aphanomyces resistance breeding programs which selected the different sources of resistance studied.

Specificity of Aphanomyces resistance meta-QTL to environments (years, locations in France and USA), strains and scoring criteria, was not observed, as discussed in [46] for QTL identified in Baccara × PI180693 and Baccara X 552 populations. In this study, we confirmed the low specificity of resistance QTL in the DSP × 90–2131 population, especially at the two highly consistent meta-QTL *MQTL-Ae16* and *MQTL-Ae25/Ae26*, towards a number of French and USA environments or a number of *A. euteiches* strains from the same or different pathotypes tested than in [46]. However, some genomic regions were still observed to be highly specific to French environments (*MQTL-Ae12, MQTL-Ae5*), controlled condition tests with different strains (*MQTL-Ae16*) or aerial scoring criteria (*MQTL-Ae27*).

### Aphanomyces resistance alleles at most the consistent QTL co-segregate with alleles at genes/QTL controlling morphological and phenological traits

In this study, we identified co-segregating alleles contributing to resistance and morphological or phenological traits in seven genomic regions, including all but one (*MQTL-Ae12*) of the seven main consistent genomic regions associated with resistance to *A. euteiches* and one less consistent resistance meta-QTL (*MQTL-Ae1*)*.* In these genomic regions, alleles derived from resistant parents were often associated with undesirable alleles for dry pea breeding (late-flowering/higher plant, normal leaves (*Af*), colored flowers (*A*); Table 2). Co-segregation between resistance and favorable alleles (round seeds (*R*), early-flowering plant), especially ones derived from 552, were identified in some genomic regions (*MQTL-Ae3, MQTL-Ae15, MQTL-Ae16/Ae17*). Marx [32] also showed that tolerance to *A. euteiches* was genetically associated with several wild-type alleles, including *A* (colored flowers) which was found to be associated with *MQTL-Ae5* in this study (*Ae-Ps2.2* in [46]), as well as *Le* (tall plants) and *Pl* (black hilum of the seeds). *Le* and *Pl* were not associated with resistance in this study, according to results obtained from one of the RIL populations studied (Baccara × PI180693, [46]). We previously reported that *R* (round seeds) and *Af* (normal leaves) were other alleles associated with Aphanomyces resistance at QTL *Aph2* and *Ae-Ps1.2, i.e. Aph3*, in pea [31,46]. In this study, we demonstrated again the positive co-segregation between *R* and resistance alleles in the DSP x 90–2131 RIL population at the *Ae-Ps5.1* QTL, i.e. *Aph2*. Fondevilla *et al.*[57] and Prioul *et al.*[58] previously reported QTL for earliness (*dfII.1, flo1* and *dfIII.2, flo2*) that we localized in same genomic regions as two main consistent QTL identified in this study for earliness and resistance to *A. euteiches* (*Flo-Ps2.2/MQTL-Ae5* on LGII and *Flo-Ps3.1/MQTL-Ae8/9* on LGIII). Co-segregation between alleles associated with late-flowering and partial resistance to *Mycosphaerella pinodes* were identified in the region on LGIII [54,55] and the same associations were observed in this study for resistance to *A. euteiches*.

### Diverse candidate genes underlie Aphanomyces resistance meta-QTL

The negative or positive associations observed in this study between resistance and morphological/phenological alleles may correspond to pleiotropic genes controlling plant architectural or developmental traits or to different closely linked genes. Pleiotropic genes controlling plant architecture or development have already been suggested as good candidates for underlying resistance QTL [2]. In pea, clusters of QTL controlling numerous traits related to plant morphology, seed protein content and yield, nitrogen nutrition, and root architecture were mapped close to architecture and developmental genes, especially *Le* and *Af*, which were shown to be localized in genomic regions having pleiotropic effects [59,60]. Our study supports the hypothesis that *Af* is a good candidate gene for pleiotropy, impacting Aphanomyces resistance (at *MQTL-Ae3*) and earliness. Evaluation of NILs or mutants at the *Af* gene [60] for resistance to *A. euteiches* will be useful for validating the pleiotropy hypothesis.

Clusters of different closely linked genes, especially resistance genes [61], located in repetitive non-coding sequences have also been reported in plants, with increasing plant genome sequences available [62,63]. In our study, several Aphanomyces resistance QTL were localized in regions previously reported to contain QTL controlling resistance to other stresses. Based on comparative mapping of SSR markers, the *MQTL-Ae8-9* (*Ae-Ps3.1*) region coincides with a main consistent resistance QTL to *M. pinodes* (*MpIII.3*, [57,58]), a major frost tolerance QTL co-localizing with the *Hr* (photoperiod high-responsive flowering) locus [64] and a minor QTL for resistance to *Fusarium oxysporum* race 2 (*Fwn3.1*, [65]). Similarly, the *MQTL-Ae15* (*Aph1*), *MQTL-Ae25/Ae26* (*Ae-Ps7.6a*) and *MQTL-Ae20* (*Ae-Ps6.1*) were identified in similar regions as the major QTL *Fwn4.1* for resistance to *F. oxysporum* race 2 [65], a major resistance QTL to *Fusarium solani*[66], along with the *Qruf* QTL for resistance to *Uromyces fabae*[67] and the QTL *FRR3* for resistance to *F. solani*[68], respectively. Regions of resistance QTL co-localizations have been suggested to underlie clusters of resistance gene analogs [2]. Especially, the main-effect *MQTL-Ae25/Ae26* region has been located in a region of RGA clusters [69], suggesting that defeated resistance gene(s) may underlie the QTL. However, other mechanisms may also be suggested for resistance QTL, i.e. defense, signal transduction, as highlighted by the diversity of candidate genes identified in this study for main meta-QTL. Our results do not favor one particular hypothesis for molecular basis of resistance QTL rather than another, corroborating Ballini *et al.* and Danan *et al.*’s conclusions [22,23]. Fine mapping and mutants studies, as well as genome sequencing efforts, will be necessary to discover causal genes underlying resistance meta-QTL to *A. euteiches* in pea and to validate hypotheses currently proposed in this study regarding co-localizations between candidate genes and resistance QTL.

## Conclusion

This study describes alleles that significantly contribute to resistance to *A. euteiches* and their positive or negative associations with morphological/phenological traits. From the meta-analysis conducted in this study, a choice of alleles at meta-QTL corresponding to seven highly consistent genomic regions controlling resistance can be recommended, with their associated markers, for use in MAS (Table 3). Resistance alleles at the most consistent region, *MQTL-Ae25/Ae26* on LGVII, and especially the high-effect allele from 90–2131, appear to be the best choices for improving resistance. Resistance allele derived from 90–2079 at *MQTL-Ae15* (*Aph1*, [31]) will be useful only in some USA environments from which the QTL was specifically detected. Resistance alleles at the five other genomic regions could be prioritized for use in MAS, depending on selection focus and breeding objectives (*i.e.* multiple-environment effects, markers, confidence intervals, association with unfavorable alleles). RILs from the DSP x 90–2131 population carrying 90–2131 alleles at most of markers linked to the main meta-QTL on LGIII, LGV and LGVII (Table 3) have been recently released [42] and will be useful in breeding for resistance to *A. euteiches*.

**Table 3:** Information about the seven highly consistent genomic regions useful for MAS

**Meta-QTL (other names)**	**LG**	**Consistent resistance enhancing allele**	**Number of variables for which allele contributed to resistance**	**Effects on environments, strains**^**a**^	**R**^**2 **^**min-max**^**b**^	**Confidence interval (cM)**^**c**^	**Recommended markers for MAS associated with resistance allele**^**d**^	**Unfavorable morphological/phenological alleles co-segregating with resistance alleles detected in this study**
MQTL-Ae3 (*Ae-Ps1.2, Aph3*)	I	552	7	Field (RI-DI), RRI-ADI	8-14%	4 - 55	D21*(270)*, AF016458 *(180)*, AC75 *(280)*	normal leaves
		PI180693	5	Field (RI-DI-LS), RRI-ADI	4-12%			normal leaves
		90-2079	3	CC (SP7, Ae106)	9-15%			normal leaves
MQTL-Ae5/Ae6 (*Ae-Ps2.2*)	II	PI180693	8	Field (RI-DI), ADI-DW CC (Ae109)	7-27%		AA372.1 *(260)*, AB112 *(390)*, AD83*(280)*, AB33 *(360)*	colored flowers, late flowering
		552	3	Field (DI), RRI CC (RB84, Ae109)	6-8%	35 - 44		
MQTL-Ae8/Ae9 (*Ae-Ps3.1*)	III	PI180693	13	Field (RI-DI-LS-ATH), RRI-ADI	8-27%	22 - 54	AA175 *(280)*, AB92 *(360)*, AD57 *(320)*	late flowering
		552	10	Field (RI-DI-PLM), RRI-ADI CC (RB84, Ae109)	6-14%			**-**
MQTL-Ae12 (*Ae-Ps4.1*)	IV	552	7	Field (RI-DI), RRI-ADI	8-21%	6 - 42	AD186 *(320)*, AA174 *(450)*	**-**
		90-2131	3	Field (RI), RRI-ADI	8-13%			**-**
		PI180693	3	Field (RI-DI), RRI-ADI	5-8%			**-**
MQTL-Ae15 (*Ae-Ps4.4, Ae-Ps4.5, Aph1*)	IV	90-2079	8	Field (PLM-LS), RRI-ADI-DWL CC (SP7, Ae106)	6-44%	8 - 28	AC22 *(200)*, AC32 *(270)*	**-**
MQTL-Ae16/Ae17 (*Ae-Ps5.1, Aph2)*	V	90-2131	7	Field (LS), ADI CC (RB84, Ae78, Ae85, Ae87 = SP7, Ae106, Ae109)	7-38%	27-36	AA81 *(250)*, AB23 *(390)*, AD68 *(290)*, AB47 *(330)*	**-**
		90-2079	4	Field (PLM, LS) CC (Ae106)	7-24%			**-**
MQTL-Ae25/Ae26 (*Ae-Ps7.6a*)	VII	90-2131	28	Field (RI-DI-TPX-PLM-LS), RRI-ADI CC (RB84, Ae78, Ae85, Ae106, Ae109)	6-60%	39 - 54	AD186 *(320)*, AA174 *(450)* 27–36 AA81 *(250)*, AB23 *(390)*, AD68 *(290)*, AB47 *(330)* 39 - 54	**-**
		PI180693	15	Field (RI-DI-LS-ATH), RRI-ADI-DW CC (RB84)	6-14%		AA505 *(180)*, AB27 *(115)*, AB136 *(330)*, AB122 *(330)*, AA387 *(470)*, AB101 *(370)*	**-**
		552	10	Field (RI-DI-PLM), RRI-ADI CC (Ae109)	6-20%			**-**

^a ^Field locations/resistance evaluation criterion are coded as followed : RI = Riec/Belon (FR); DI = Dijon (FR), TPX = Templeux (FR), PLM = Pullman (USA); LS = LeSueur (USA); RRI = Root Rot Index; ADI = Aerial Decline Index; DW = Dwarfism; DWL =% Dried Weight Losses. Controlled-condition scoring traits are indicated by the name of the *A. euteiches *strain (RB84, Ae78, Ae85, Ae87 = SP7, Ae106 and Ae109).

^b ^Range of percentage of phenotypic variation explained by each resistance-enhancing allele at each meta-QTL, depending on the variable.

^c ^Confidence interval of the single or two meta-QTL region (1st value) and of the genomic region encompassing LOD-1 confidence intervals of all initial QTL underlying the meta-QTL (2nd value), in cM Kosambi.

^d ^Recommended co-dominant SSR markers covering the genomic region encompassing LOD-1 confidence intervals of all initial QTL underlying the meta-QTL. Mean allele sizes are indicated *in parentheses* for each SSR.

Because sources of resistance are limited [35] and the number of highly consistent resistance QTL highlighted from this study is moderately low, the most consistent genomic regions controlling resistance should be carefully managed to prevent reduced effectiveness due to potential development of new virulent isolates of *A. euteiches*. In alfalfa, strains of *A. euteiches* from race 2 have been reported to overcome resistance to race 1 in cultivars grown in the USA [70]. In *Medicago truncatula*, the level and nature of the effect of a major Aphanomyces resistance QTL (*AER1/prAe1*, [71,72]) have been shown to vary depending on the pathotypes of *A. euteiches* strains [73], suggesting that QTL might have variable effects on resistance depending on the evolution of *A. euteiches* strains. Despite the limited potential for gene flow in soilborne pathogens, *A. euteiches* could be assigned to the group of medium-moderately high evolutionary risk pathogens (scale 5–7) [8], since the genetic structure of *A. euteiches* populations was reported to be patterned by a mixed reproduction system, including regular selfing with occasional migration of novel genotypes or outcrossing [74]. Consequently, pyramiding consistent Aphanomyces resistance QTL is strongly recommended, both for further increasing levels of resistance and for attempting to preserve the durability of each QTL effect. The best combinations of QTL to increase resistance efficiency and durability have yet to be determined. Validation of QTL effects in different genetic backgrounds, knowledge of QTL effects on pathogen life cycle and epidemic development and an understanding of molecular mechanisms underlying QTL, will help identify QTL combinations that should be integrated in pyramiding strategies for durable resistance breeding.

## Methods

### Plant material

A population of 111 F_10_-derived pea recombinant inbred lines from the cross Dark Skin Perfection × 90–2131 was produced by single-seed descent at USDA-ARS, Pullman, WA, USA. DSP (Dark Skin Perfection, Unilever Limited 1960, also designated as W6 17516) is a spring pea cultivar used for freezing and canning, with white flowers, normal leaves, wrinkled seeds with white hilum and it is susceptible to *A. euteiches*. 90–2131 [38] is a pea germplasm with white flowers, normal leaves, dimpled seeds with black hilum and it is partially resistant to *A. euteiches* in France and in the USA. The parental lines, 90–2131 and DSP, were used as check lines in field- and controlled-condition disease resistance tests of the DSP × 90–2131 RIL population. The three RIL parental lines previously studied in [46], Baccara (susceptible), PI180693 and 552 (partially resistant), were also used in the controlled condition assays. The MN313 line was included in the assays for distinguishing the two main pea pathotypes of *A. euteiches*[51]. The pea spring variety Solara (susceptible) was used as the adjacent control in the field assays of the RIL population.

### Pathogen material

Six pure culture strains of *A. euteiches* virulent on pea were used for resistance evaluation of the DSP × 90–2131 RIL population in controlled conditions. These strains were RB84, Ae106, Ae78, Ae85, Ae87 (referred to as strain SP7 in [51]) and Ae109 (referred to as strain Ae467 in [75] and [33]). The six strains were chosen based on their various geographical origins and pathotype groups (Additional file 1). The strains Ae106 and Ae87, were previously used for disease screening of the Puget x 90–2079 RIL population [45] and the strains RB84 and Ae109 were used in the Baccara × PI180693 and Baccara x 552 RIL populations screenings [46].

### Disease resistance evaluations

In controlled conditions, resistance of the DSP × 90–2131 RILs to pure-culture strains of *A. euteiches* was assessed on 14-day-old seedlings in a climatic chamber, as described in [46], with one strain and four blocks per test. Each block included all RILs and control lines, with five plants/line/block. A root rot index (RRI) ranging from 0 to 5 was calculated as the mean disease severity score on the five plants per line, as described in [46].

In the field, the DSP × 90–2131 RILs were evaluated over an international Aphanomyces infested nursery network, described in [46], over four years (2000 to 2003) and five locations in France (Riec-sur-Belon, Finistère (RI); Dijon-Epoisses, Côte d’Or (DI); Templeux, Somme (TPX)) and in the United States (Pullman, WA (PLM); LeSueur, MN (LS)) [in 2000: PLM, LS; in 2001: LS; in 2002 : RI, DI, LS; in 2003: RI, DI, TPX, PLM, LS]. Field assays were carried out using experimental designs as described in [46]. For field experiments conducted in France (two years, three locations) and in Pullman in 2003, the design included a check plot of the susceptible cultivar Solara or DSP every two to four plots, in order to adjust disease severity scores for local disease variations in the soil [76] using the formula described in [46,77]. Two disease criteria were used to assess resistance for each plot, as described in [46]: (i) the root rot index (RRI), using a 0–5 scoring scale, evaluated each year in French nurseries, except at DI in 2002, and (ii) the aerial decline index (ADI) evaluated once, twice or three times in all USA and French disease nurseries (except at RI in 2002), using a 1–5 and 1–9 disease scoring scale, respectively.

### Evaluation of morphological and phenological traits

The DSP × 90–2131, Baccara × 552 and/or Baccara × PI180693 RIL populations were evaluated for two agronomic traits: earliness at flowering (FLO) and plant height (HT).

The FLO trait was evaluated in the Aphanomyces-infested nursery of DI in 2003 (DSP × 90–2131 RILs), 2004 (Baccara × 552 RILs), 2006, 2007 and 2008 (Baccara × PI180693 RILs) using the experimental design established for resistance evaluation. The FLO trait was also evaluated in a healthy nursery at Rennes-Le Rheu (Ille-et-Vilaine, FR (REN)) in 2002 (DSP × 90–2131 RILs), 2005 and 2008 (Baccara x PI180693 RILs) using a randomized complete block design with 1 and 3 block(s) in 2002/2005 and 2008, respectively (40 plants/plot in a two m-long twin rows). The FLO trait was scored on each plot as the number of days to 50% bloom (FLO1) or to 100% bloom (FLO2) from the first day of the year. The HT trait was evaluated in the same REN healthy nurseries as used for FLO evaluation in 2002 (DSP × 90–2131 RILs), by measuring the average height of five plants at maturity in a whole plot.

### Molecular markers and genetic mapping

The DSP × 90–2131 RIL population was genotyped using simple sequence repeat (SSR) from [50], random amplified polymorphic DNA (RAPD) [78] and known-function genes [48,49]. Two morphological traits were also scored: *Pl* for hilum colour and *R* for round/wrinkled seeds. DNA extractions and PCR amplifications were performed as described in [46] and in [48,49,53]. The Puget × 90–2079 RIL population was genotyped using additional SSR, RAPD, SCAR and known-function gene markers from [49,53,69,78-80] compared to markers reported in [31]. Marker coding and the genetic map (in cM Kosambi) from the DSP × 90–2131 RIL population were established with a minimum LOD score threshold of 3.0 and a maximum recombination frequency of 0.4, as described in [46]. For each locus, adjustment of allelic segregation to the expected 1:1 Mendelian ratio was analyzed using a χ^2^ test (α=0.01). Additional markers were placed on the framework Puget × 90–2079 genetic map reported in [31], using the “assign” and “place” commands of MAPMAKER/EXP version 3.0b [81].

### Statistical and QTL analyses

Statistical and QTL analyses were conducted from field and controlled-condition disease scoring data obtained in the DSP × 90–2131 RIL population and from earliness and plant height data obtained in the DSP × 90–2131, Baccara × PI180693 and Baccara × 552 RIL populations, for each scoring variable in each environment. Statistical analysis of each data set was carried out using a two-way ANOVA estimating genotype and block effects and normality of residuals was analyzed, as described in [46]. Broad sense heritability, RIL least-square means used for linkage analysis and Pearson correlation coefficients (*r*^*2*^) between adjusted mean data were also estimated as described in [46].

Additive-effect QTL analysis from each data set was performed by Composite Interval Mapping [82] using Windows QTL Cartographer 2.5 software [83], as described in [46]. Using the permutation test with 1000 permutations, minimum LOD thresholds of 2.9 (for DSP × 90–2131 RIL population) and 2.8 (for the Baccara × PI180693 and Baccara × 552 RIL populations) were chosen for all the traits to declare a putative QTL significant, corresponding to a genome-wide α error risk of 5%. Two QTL were considered as belonging to the same genomic region when their one-LOD drop-off confidence intervals overlapped. QTL for resistance to *A. euteiches*, earliness and plant height were named “*Ae-Ps*”, “*Flo-Ps*” and “*HT-Ps*”, respectively, followed by the linkage group number and the QTL number within the linkage group for each trait. Based on common markers between genetic maps, Aphanomyces resistance QTL common to those previously published [31,46] were named as described in [46]. The most significant pairwise epistatic interactions were detected for each resistance variable between all possible marker pairs of the DSP × 90–2131 genetic map, as described in [73] using a detection threshold of *P* < 7,1.10^-6^ and *R*^2^ > 5%.

From the Puget × 90–2079 RIL population, additive-effect QTL were re-detected, as described in [31], from the updated genetic map generated in this study and the phenotypic data reported in [31,45].

### QTL meta-analysis

Using all the additive-effect QTL identified for resistance to *A. euteiches*, earliness and plant height from Puget × 90–2079 [31,78], Baccara × PI180693, Baccara x 552 [46] and DSP × 90–2131 (this study) RIL populations, a QTL meta-analysis was performed using the MetaQTL software version 1.0 [17].

QTL meta-analysis was conducted in three steps, according to details given in [21]. For each QTL, data given to the software were the QTL position (LG, position on the LG at the LOD peak), the upper and lower bound and LOD decrease of the QTL confidence interval, the percentage of variation (*R*^*2*^*)* individually explained by the QTL, the trait related to the QTL and the size of the mapping population used for the QTL detection. First, a single consensus marker map was built by integrating the available genetic maps, based on common markers designated with common names between the maps, using the *ConsMap* command of the software. The implemented method applied a weighted least-square strategy, using individual distances between markers in each individual map, to determine marker order and position on the consensus marker map. The *InfoMap* command was carried out in order to list markers whose orders were not consistent between the different individual maps. Markers with inconsistent positions were removed from the consensus marker map.

Second, the QTL detected in each study were projected onto the consensus map, using the *QTLproj* command of the software. This command enabled the homothetic projection of the individual QTL positions and confidence intervals based on a scaling rule between QTL-flanking marker positions on the individual maps and on the consensus map. QTL projection was carried out using LOD-1 confidence intervals of all individual QTL, for graphical representation and for identifying main genomic regions comprised of overlapping QTL intervals. For the meta-analysis, the projection was carried out using independent individual QTL, since the QTL meta-analysis algorithm implemented in the software assumes that the input mapping studies are independent from each other. Independent QTL were selected as follows. In a given mapping population and for a given variable, when QTL detected from various environments or strains had overlapping confidence intervals, only the QTL with the greatest proportion of the phenotypic variation (*R*^2^) and the smallest confidence interval (if highest *R*^2^ were equals) was retained. For example, among the five individual QTL detected at the top of LGII for the ADI variable in different environments (RI04, RI05, PLM04, TPX04) in the Baccara x 552 population, the one having the greatest *R*^*2*^ (15%) was kept for the meta-analysis and the four others were removed. Confidence intervals (CI) of independent QTL were estimated from the QTL *R*^2^ values, using the empirical formula proposed by [55]: $CI=\frac{530}{\text{NxR²}}$, where N is the population size. This formula usually gives larger confidence intervals than the usual interval length of LOD-1 decrease.

Finally, the QTL meta-analysis algorithm, computed in the *QTLClust* command of the software, was used to determine the most likely number of meta-QTL on a given chromosome and to estimate their corresponding positions and confidence intervals. Meta-QTL considers QTL positions and corresponding confidence intervals in individual experiments, after projection onto the consensus map. We used the *--cimode* option 4 of *QTLClust* command that consider the maximum confidence interval between the LOD-1 decrease value reported and the QTL *R*^*2*^ value estimated by the formula of [55]. On a given chromosome, projected independent QTL were clustered into all possible numbers of hypothetic clusters of meta-QTL (*K*), for which a Gaussian mixture model estimates meta-QTL positions and confidence intervals [17]. The optimal *K* was determined using the highest weight of evidence values estimated for five information-based criteria [17], which were computed for each *K*. The most frequent optimal *K* value given by the different criteria was selected, which always corresponded to the optimal *K* value determined by the Akaike Information Criterion. The position, 95% confidence interval and probability of individual QTL belonging to the meta-QTL, were given by the software for each meta-QTL.

Two meta-analysis runs were conducted separately for Aphanomyces resistance QTL data and earliness and plant height QTL data, respectively, resulting in the detection of meta-QTL for two types of traits, which were then compared.

Meta-QTL for resistance to *A. euteiches* and meta-QTL for earliness and plant height were named “*MQTL-Ae*” and “*MQTL-Morpho*”, respectively, followed by the meta-QTL number on the whole pea genome.

### Identification of candidate genes underlying meta-QTL

Positional candidate genes included within the support interval of main resistance meta-QTL regions were identified using the Pea-*M. truncatula* toolkit of Bordat *et al.*[47]. Mining the high colinearity between the pea and *M. truncatula* genomes, the toolkit allowed placing *in silico* 5460 pea Unigenes on the pea consensus map of [47], from positions of their best homologs on the *M. truncatula* genome. Positions of main meta-QTL regions identified in this study were estimated on the pea consensus map of [47] based on common markers, mainly SSRs. On the pea map of [47], intervals between adjacent marker genes covering main meta-QTL regions were identified and a list of Unigenes contained in these intervals was established. Intervals defined from adjacent marker genes genetically mapped in [47] were often larger than the reduced confidence interval of meta-QTL or absent. In these cases, intervals at most probable meta-QTL positions were chosen. Positional candidate genes were examined to identify those known to be involved in disease resistance in plants.

## Competing interests

The authors declare that they have no competing interests.

## Authors’ contributions

CH carried out QTL meta-analysis and genetic analysis from the RIL populations and contributed to drafting the manuscript. CC participated in the design of the study (construction of the Puget x 90–2079 and DSP x 90–2131 RIL populations, of the multi-site USA field Aphanomyces networks) and contributed to molecular and field data acquisition. RM, RE, PM, MR-D and KM designed and carried out field Aphanomyces assays from the multi-site USA-FR Aphanomyces field network and participated in substantial field data acquisition. AL coordinated, carried out and analyzed Aphanomyces resistance data obtained in controlled conditions. MH, IL and GD carried out substantial genotyping of RIL populations and contributed to the establishment of individual genetic maps. GM coordinated plant material seed increase and distribution. RD participated in coordination of the study as thesis director of CH. AB participated in the design and coordination of the study and revised the draft manuscript critically. MLP-N conceived of the study, headed its design and coordination and contributed to drafting the manuscript. All authors read and approved the final manuscript. CC, RM and KM critically revised the manuscript, especially its English written style.

## Supplementary Material

Additional file 1 **Strains of *Aphanomyces euteiches *used, with code number, geographical origin and pathotype group. **^a ^The pathotype groups of *A. euteiches *strains were described in Wicker and Rouxel (2001) and defined by their differential reactions on a six pea genotypes (Wicker *et al. *2003). Click here for file

Additional file 2 **Frequency distribution of adjusted means of root rot index (RRI) scores for resistance to six strains of *A.euteiches, *in the DSP x 90–2131 pea RIL population. **Values of the partially resistant (90–2131) and susceptible (DSP) parents, named P_R _and P_S_, respectively, are shown by arrows. ^*a*^n = total number of RILs assessed; ^*b*^m = mean ± standard deviation of the RIL population; ^*c*^h^2^ = heritability estimate. Strains: (a) RB84 (b) Ae106 (c) Ae85 (d) Ae87 (e) Ae78 (f) Ae109. Click here for file

Additional file 3 **Frequency distributions of adjusted mean scores obtained in the DSP x 90–2131 RIL population for two Aphanomyces root rot resistance criteria (root rot and aerial decline indexes) assessed in 11 environments over four years and five locations in France and the USA. **In each environment, one to three ADI scores were obtained. Adjusted mean values of the partially resistant (90–2131) and susceptible (DSP) parents, named P_R _and P_S_, respectively, are shown by arrows. ^*a*^n = total number of RILs assessed; ^*b*^m = mean ± standard deviation of the RIL population; ^*c*^h^2^ = mean-based heritability of the trait. Click here for file

Additional file 4 **Pearson correlation coefficients between the different adjusted mean scoring data obtained for Aphanomyces root rot resistance in the DSP x 90–2131 RIL population in 11 field environments and in controlled conditions with RB84, Ae106, Ae85, Ae87, Ae78 and Ae109 strains of *A. euteiches. ***Significance level threshold (P < 0.001) are indicated by asterisks (***). Field scoring variables are coded with abbreviations of the locations, years and scoring criteria tested: RI = Riec/Belon (FR); DI = Dijon-Epoisses (FR); TPX = Templeux (FR); PLM = Pullman (USA); LS = LeSueur (USA); 00 = 2000; 01 = 2001; 02 = 2002; 03 = 2003; RRI = Root Rot Index; ADI = Aerial Decline Index. Click here for file

Additional file 5 **Additive-effect QTL identified from the DSP x 90–2131 RIL population for resistance to *A. euteiches *in infected fields over 11 environments in France and in the USA, and in controlled conditions using six pure-culture strains of *A. euteiches. ***Values of QTL parameters were obtained using Windows QTL Cartographer 2.5 (LOD ≥ 2.9). The QTL are ordered by position on the LG. ^a^ Based on common markers, QTL common to Hamon *et al. *(2011) were named as they were previously. ^b ^Field scoring traits are coded as follows: location (RI = Riec/Belon (FR); DI = Dijon (FR), TPX = Templeux (FR), PLM = Pullman (USA); LS = LeSueur (USA)); year (2000 = 00; 2001 = 01; 2002 = 02; 2003 = 03); criterion (RRI = Root Rot Index; ADI = Aerial Decline Index). Controlled-condition scoring traits are indicated by the name of the strain (RB84, Ae78, Ae85, Ae87, Ae106 and Ae109). ^c ^QTL position from the first marker of the linkage group (in centimorgans Kosambi) ^d^ Nearest left marker from the LOD score peak of the QTL ^e ^Log of likelihood ratio (LOD) value at the LOD peak of the QTL for each variable ^f^ Position of the lower and upper of the one-LOD drop-off confidence interval of the QTL, from the first marker of the linkage group (in centimorgans Kosambi) ^g^ Percentage of phenotypic variance explained by an individual QTL ^h ^Effect of substituting DSP alleles for 90 2131 alleles at the QTL. A positive sign indicates that QTL alleles increasing the resistance are contributed by the resistant parent 90–2131, whereas a negative sign means that resistant alleles are brought by the susceptible parent DSP. Click here for file

Additional file 6 **Pairwise epistatic interactions associated with resistance to Aphanomyces root rot, identified from the DSP x 90–2131 RIL populations (P < 7,1.10**^**-6**^**). **QTL are ordered by QTL name and decreasing R^2 ^values. ^a ^The name of additive-effect QTL involved in epistatic interactions are indicated in bold. ^b ^Significance level of each pairwise epistatic interaction in an ANOVA model with two factors (markers) and an interaction factor (interaction between two markers) ^c ^Percentage of phenotypic variation explained by each individual interaction ^d ^Adjusted mean scores for the four genotypic classes defined by each marker pair : (SS) and (RR) DSP and 90–2131 alleles at the two markers, respectively; (SR) DSP allele at the first marker, 90–2131 allele at the second marker and conversely (RS). In bold: the most resistant genotypic class. Click here for file

Additional file 7 **Frequency distributions of adjusted mean scores obtained in the DSP x 90–2131, Baccara x 552 and Baccara x PI180693 RIL populations for plant height (HT, in cm) and flowering time (Flo1 and Flo2, in number of days from the first day of the year).** Adjusted mean values of the partially resistant (90–2131, PI180693 or 552) and susceptible (DSP or Baccara) parents, named P_R _and P_S_, respectively, are shown by arrows. ^*a*^n = total number of RILs assessed; ^*b*^m = mean ± standard deviation of the RIL population; ^*c*^h^2^ = mean-based heritability calculated when scores were obtained on three blocks. Click here for file

Additional file 8 **Additive-effect QTL identified from the DSP x 90–2131, Baccara x PI180693 and Baccara x 552 RIL populations for flowering time and plant height traits, scored in eight infested or healthy environments (values obtained by Windows QTL Cartographer 2.5, LOD ≥ 2.9 for the DSP x 90-2131 RIL population and LOD ≥ 2.8 for the Baccara x PI180693 and Baccara x 552 RIL populations). **The QTL are ordered by position on the LG. ^a ^Scoring traits are coded as follows: population (D2131 = DSP x 90 2131; B552 = Baccara x 552; BPI = Baccara x PI180693); location (DI = Dijon (FR); REN = Rennes (FR)); year (02 = 2002; 03 = 2003; 04 = 2004; 05 = 2005; 06 = 2006; 01 = 2007; 08 = 2008); criterion (Flo1 and Flo2 = Flowering times; HT = Plant Height) ^b ^QTL position from the first marker of the linkage group (in centimorgans Kosambi) ^c^ Nearest left marker from the LOD score peak of the QTL ^d ^Log of likelihood ratio (LOD) value at the LOD peak of the QTL for each variable ^e^ Position of the lower and upper of the one-LOD drop-off confidence interval of the QTL, from the first marker of the linkage group (in centimorgans Kosambi) ^f^ Percentage of phenotypic variance explained by each individual QTL ^g^ Effect of substituting Baccara or DSP alleles for 552 or PI180693 or 90-2131 alleles at the QTL. A positive sign indicates that QTL alleles increasing the earliness at flowering or decreasing plant height are contributed by the resistant parent 552, or PI180693 or 90-2131, whereas a negative sign means that earliness or short plant alleles are brought by the susceptible parent Baccara or DSP. Click here for file

Additional file 9 Localization of individual QTL and meta-QTL for resistance and morphological traits onto the consensus marker map: linkage group VII (for legend, see Figure 2). Click here for file

Additional file 10 **Description of the pea consensus marker map established using the three individual genetic maps (Baccara x PI180693/552, DSP x 90–2131 and Puget x 90–2079) using the MetaQTL software. **Cumulated genetic distances between markers are indicated in centimorgans Kosambi. The occurrence number of each marker across the three individual maps is indicated (Meta occurrence). Click here for file

Additional file 11 **List of putative candidate genes underlying main Aphanomyces resistance meta-QTL regions, identified using the pea-*M. truncatula *translational toolkit **[47]**. **Candidate genes and their contig sequence names were identified from positions of adjacent gene markers mapped on the consensus genetic map of [47], which were estimated to be located in the same regions as the meta-QTL by comparative mapping. Click here for file
