# Supplementary material for: QTL meta-analysis provides a comprehensive view of loci controlling partial resistance to Aphanomyces euteiches in four sources of resistance in pea
Source: BMC Plant Biol. 2013 Mar 16;13:45. doi: 10.1186/1471-2229-13-45 (PMC3680057; doi:10.1186/1471-2229-13-45)
Supplement: Additional file 1 — Strains of Aphanomyces euteiches used, with code number, geographical origin and pathotype group. a The pathotype groups of A. euteiches strains were described in Wicker and Rouxel (2001) and defined by their differential reactions on a six pea genotypes (Wicker et al. 2003). [file 1471-2229-13-45-S1.pdf]

Code	Country (Region)	Pathotype group[a]	References
RB84	France (Brittany)	I	Moussart et al. (2007)
Ae106	France (Eastern Parisian Basin)	I	Wicker and Rouxel (2001)
Ae85	France (Eastern Parisian Basin)	I	Wicker and Rouxel (2001)
Ae87 (SP7)	USA (Idaho)	I	Kraft et al. (1994)
Ae78	France (Eastern Parisian Basin)	II	Wicker and Rouxel (2001)
Ae109 (467)	USA (Wisconsin)	III	Malvick and Percich (1998)
