# Supplementary figures and images for: QTL meta-analysis provides a comprehensive view of loci controlling partial resistance to Aphanomyces euteiches in four sources of resistance in pea
Source: BMC Plant Biol. 2013 Mar 16;13:45. doi: 10.1186/1471-2229-13-45 (PMC3680057; doi:10.1186/1471-2229-13-45)

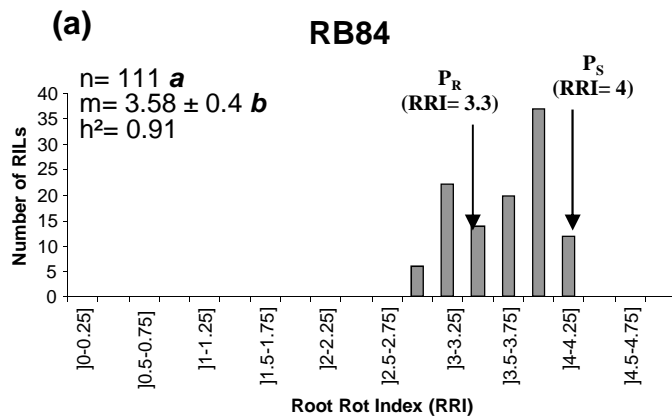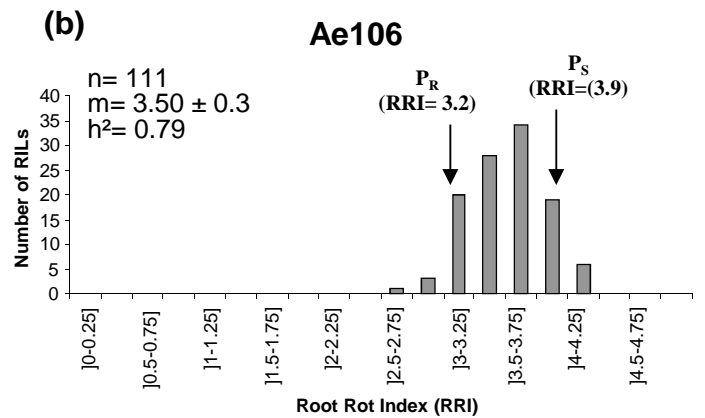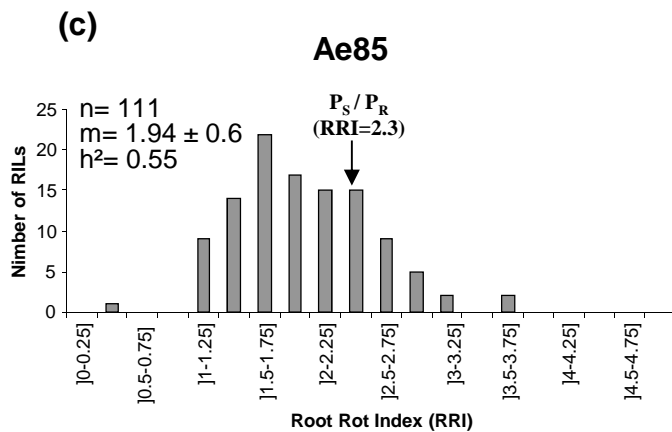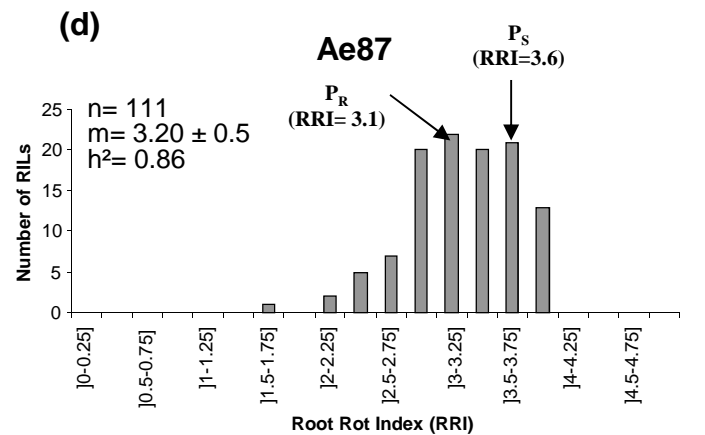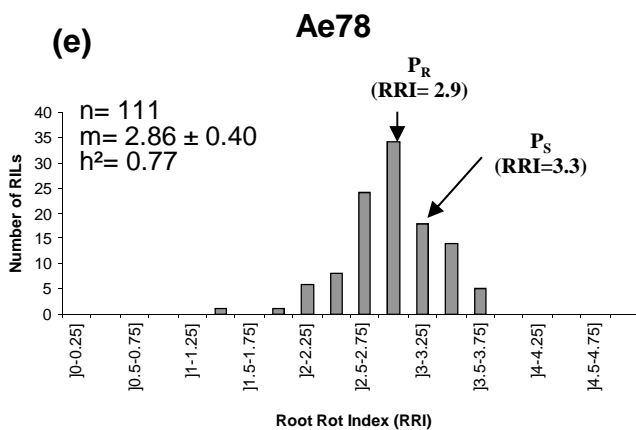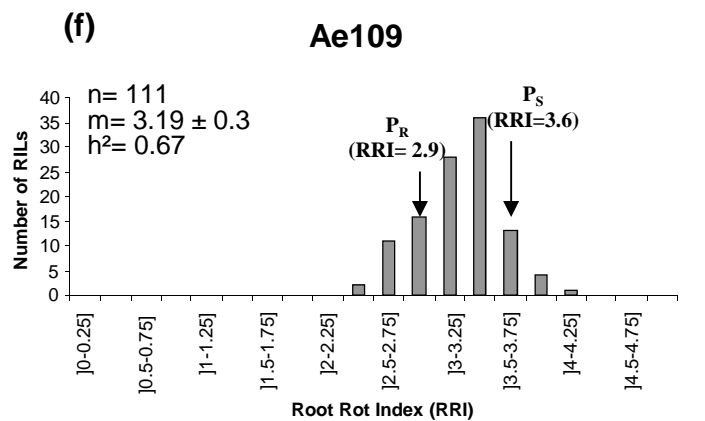

Supplement: Additional file 2 — Frequency distribution of adjusted means of root rot index (RRI) scores for resistance to six strains of A.euteiches, in the DSP x 90–2131 pea RIL population. Values of the partially resistant (90–2131) and susceptible (DSP) parents, named PR and PS, respectively, are shown by arrows. an = total number of RILs assessed; bm = mean ± standard deviation of the RIL population; ch2 = heritability estimate. Strains: (a) RB84 (b) Ae106 (c) Ae85 (d) Ae87 (e) Ae78 (f) Ae109. [file 1471-2229-13-45-S2.pdf]

(a) DSP x 90-2131 RIL population

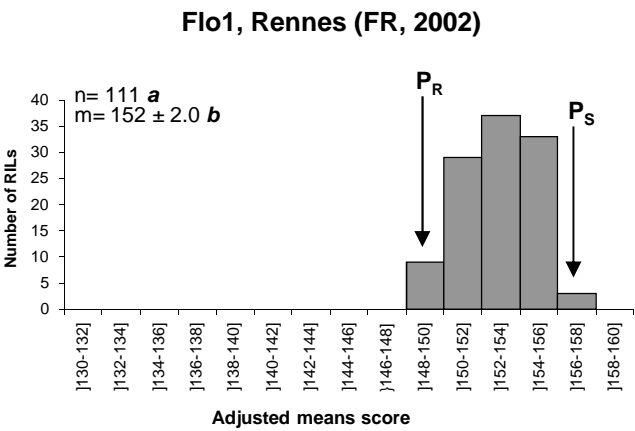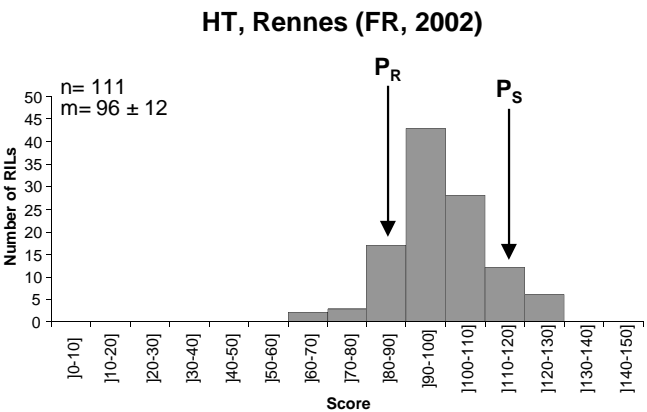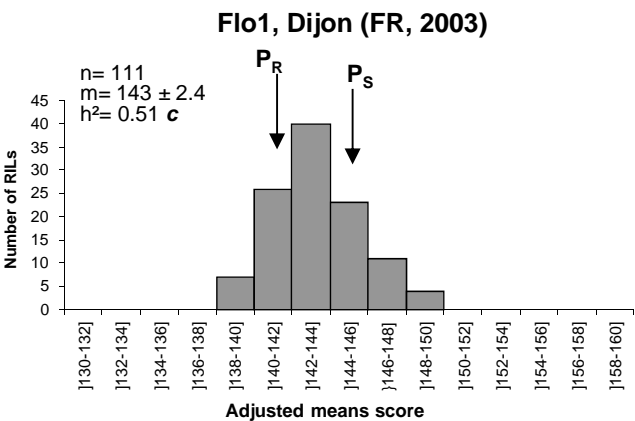

(b) Baccara x 552 RIL population

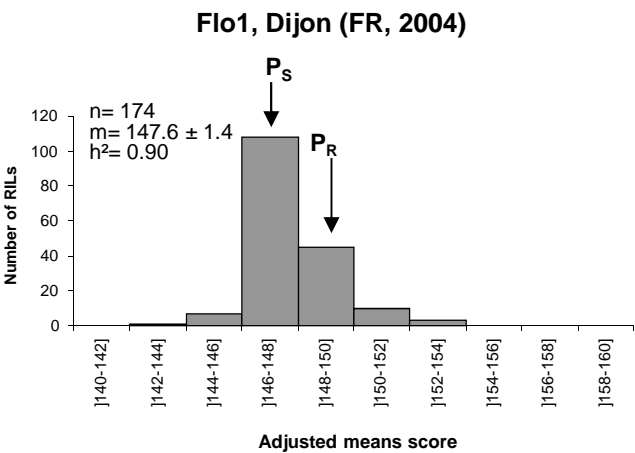

(c) Baccara x PI180693 RIL population

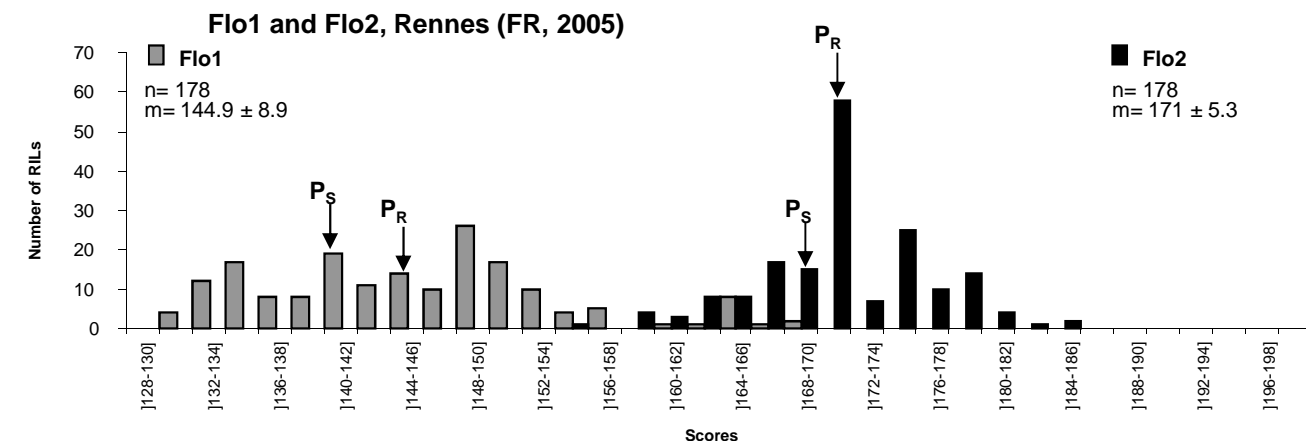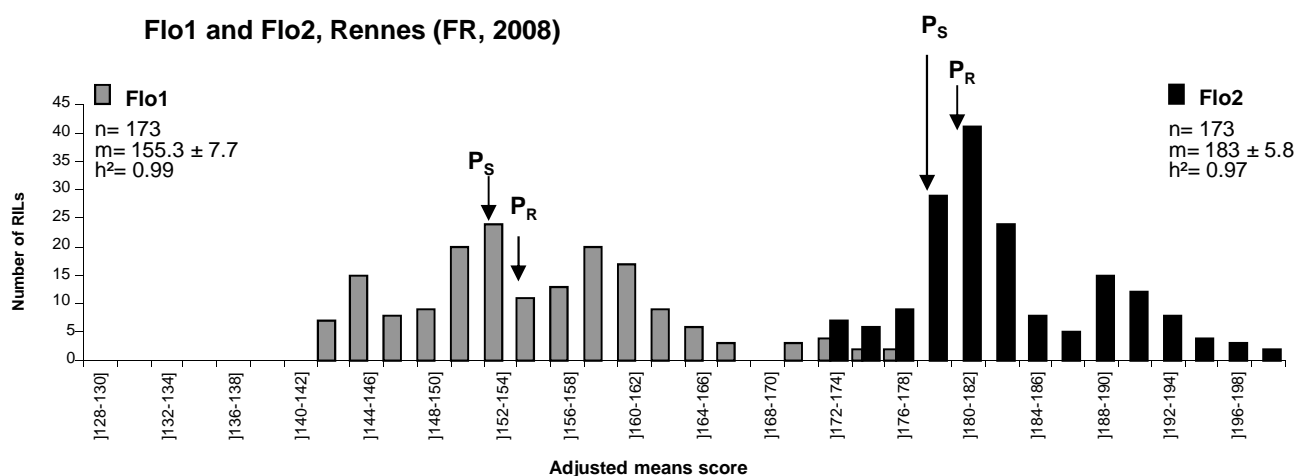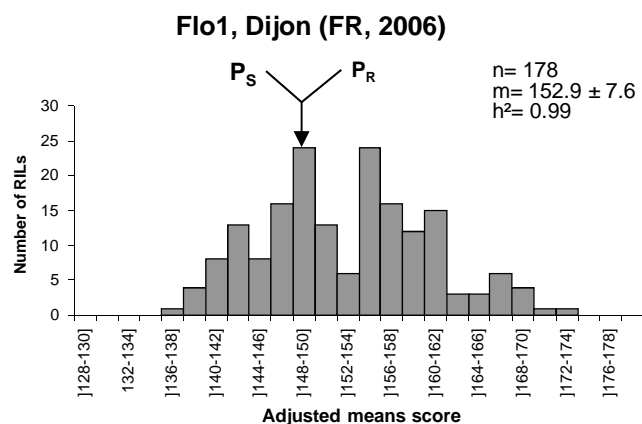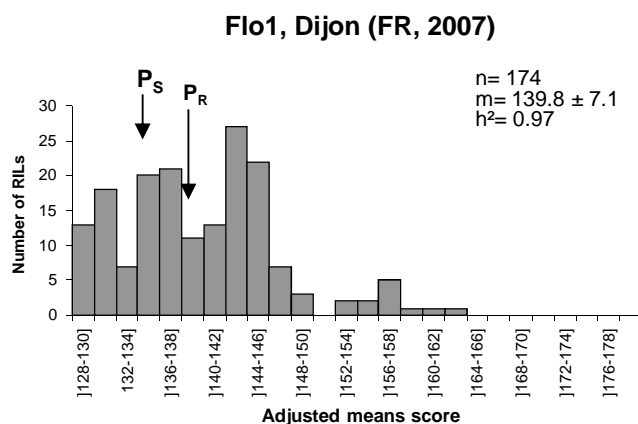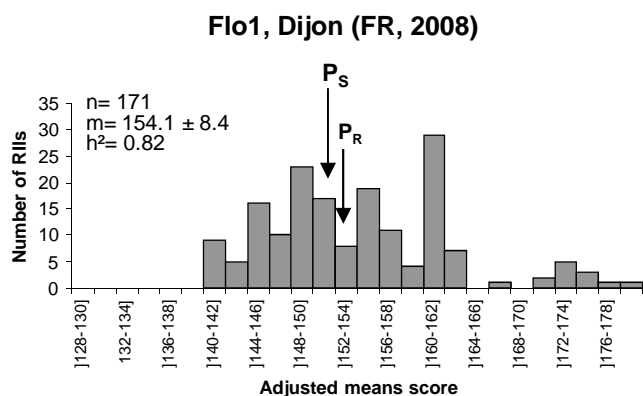

Supplement: Additional file 7 — Frequency distributions of adjusted mean scores obtained in the DSP x 90–2131, Baccara x 552 and Baccara x PI180693 RIL populations for plant height (HT, in cm) and flowering time (Flo1 and Flo2, in number of days from the first day of the year). Adjusted mean values of the partially resistant (90–2131, PI180693 or 552) and susceptible (DSP or Baccara) parents, named PR and PS, respectively, are shown by arrows. an = total number of RILs assessed; bm = mean ± standard deviation of the RIL population; ch2 = mean-based heritability calculated when scores were obtained on three blocks. [file 1471-2229-13-45-S7.pdf]
