# Supplementary material for: QTL meta-analysis provides a comprehensive view of loci controlling partial resistance to Aphanomyces euteiches in four sources of resistance in pea
Source: BMC Plant Biol. 2013 Mar 16;13:45. doi: 10.1186/1471-2229-13-45 (PMC3680057; doi:10.1186/1471-2229-13-45)
Supplement: Additional file 3 — Frequency distributions of adjusted mean scores obtained in the DSP x 90–2131 RIL population for two Aphanomyces root rot resistance criteria (root rot and aerial decline indexes) assessed in 11 environments over four years and five locations in France and the USA. In each environment, one to three ADI scores were obtained. Adjusted mean values of the partially resistant (90–2131) and susceptible (DSP) parents, named PR and PS, respectively, are shown by arrows. an = total number of RILs assessed; bm = mean ± standard deviation of the RIL population; ch2 = mean-based heritability of the trait. [file 1471-2229-13-45-S3.pdf]

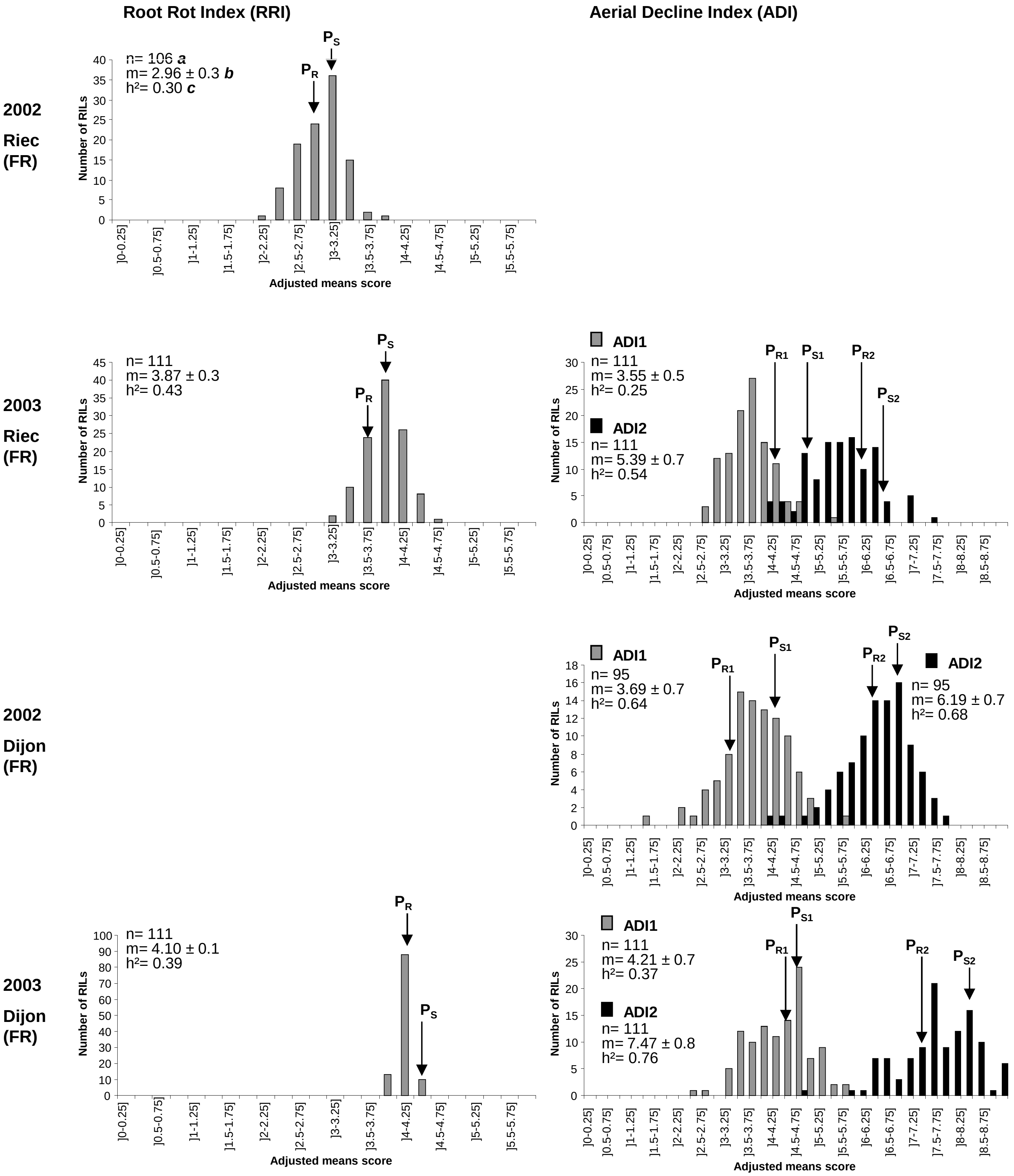
Root Rot Index (RRI)
Aerial Decline Index (ADI)
2002
Riec
(FR)
n= 106 a
m= 2.96 ± 0.3 b
h²= 0.30 c
PS
PR
Number of RILs
Adjusted means score
2003
Riec
(FR)
n= 111
m= 3.87 ± 0.3
h²= 0.43
PS
PR
ADI1
n= 111
m= 3.55 ± 0.5
h²= 0.25
ADI2
n= 111
m= 5.39 ± 0.7
h²= 0.54
PR1
PS1
PR2
PS2
2002
Dijon
(FR)
ADI1
n= 95
m= 3.69 ± 0.7
h²= 0.64
ADI2
n= 95
m= 6.19 ± 0.7
h²= 0.68
PR1
PS1
PR2
PS2
2003
Dijon
(FR)
n= 111
m= 4.10 ± 0.1
h²= 0.39
PR
PS
ADI1
n= 111
m= 4.21 ± 0.7
h²= 0.37
ADI2
n= 111
m= 7.47 ± 0.8
h²= 0.76
PR1
PS1
PR2
PS2
]0-0.25]
]0.5-0.75]
]1-1.25]
]1.5-1.75]
]2-2.25]
]2.5-2.75]
]3-3.25]
]3.5-3.75]
]4-4.25]
]4.5-4.75]
]5-5.25]
]5.5-5.75]
]6-6.25]
]6.5-6.75]
]7-7.25]
]7.5-7.75]
]8-8.25]
]8.5-8.75]

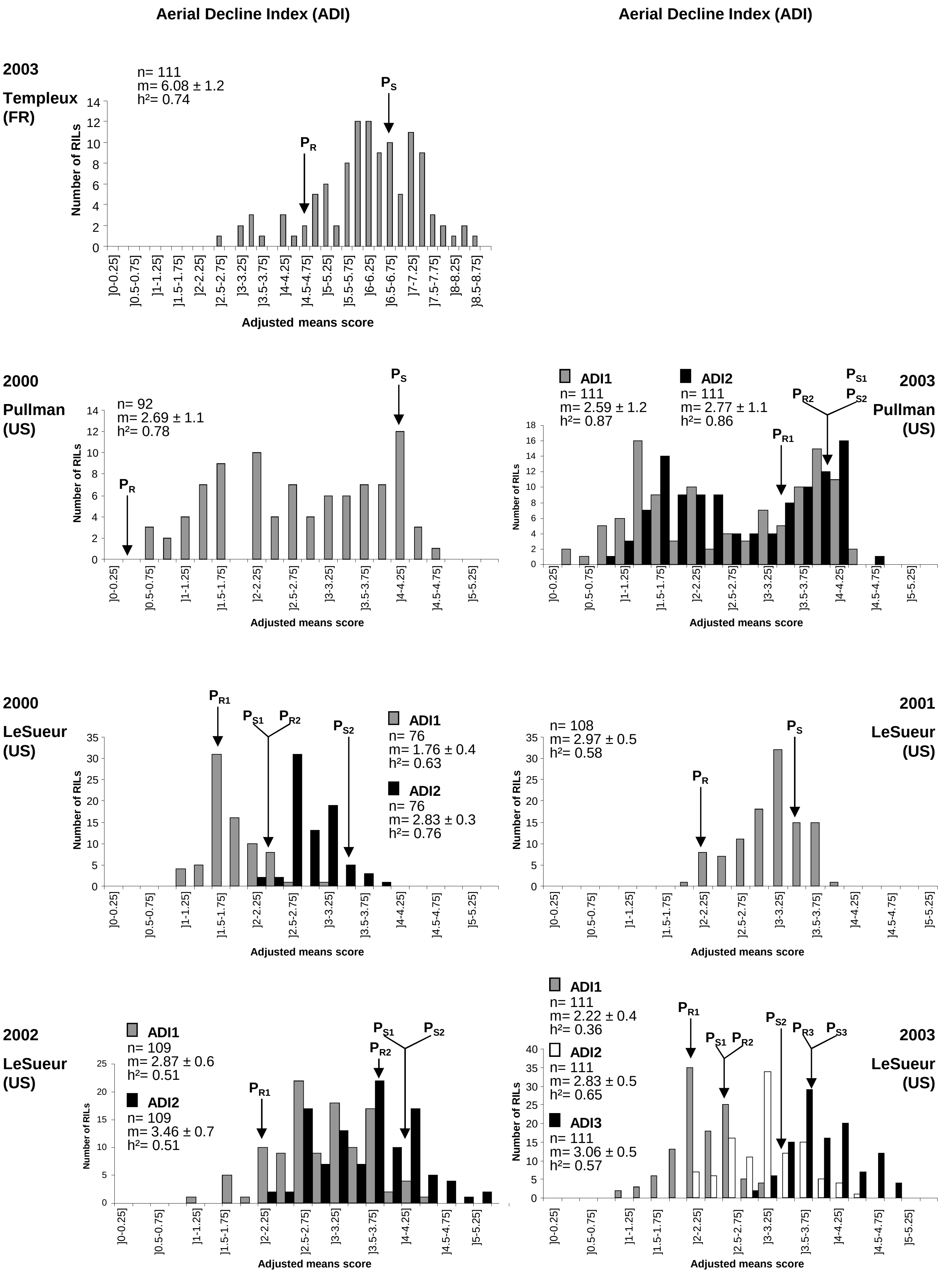
Aerial Decline Index (ADI)
Aerial Decline Index (ADI)
2003
Templeux (FR)
n= 111
m= 6.08 ± 1.2
h²= 0.74
P_S
P_R
Number of RILs
Adjusted means score
]0-0.25]]0.5-0.75]]1-1.25]]1.5-1.75]]2-2.25]]2.5-2.75]]3-3.25]]3.5-3.75]]4-4.25]]4.5-4.75]]5-5.25]]5.5-5.75]]6-6.25]]6.5-6.75]]7-7.25]]7.5-7.75]]8-8.25]]8.5-8.75]
2000
Pullman (US)
n= 92
m= 2.69 ± 1.1
h²= 0.78
P_S
P_R
Number of RILs
Adjusted means score
ADI1
n= 111
m= 2.59 ± 1.2
h²= 0.87
ADI2
n= 111
m= 2.77 ± 1.1
h²= 0.86
P_S1
P_R2
P_S2
P_R1
2003
Pullman (US)
Number of RILs
Adjusted means score
2000
LeSueur (US)
P_R1
P_S1
P_R2
P_S2
ADI1
n= 76
m= 1.76 ± 0.4
h²= 0.63
ADI2
n= 76
m= 2.83 ± 0.3
h²= 0.76
Number of RILs
Adjusted means score
2001
LeSueur (US)
n= 108
m= 2.97 ± 0.5
h²= 0.58
P_S
P_R
Number of RILs
Adjusted means score
2002
LeSueur (US)
ADI1
n= 109
m= 2.87 ± 0.6
h²= 0.51
ADI2
n= 109
m= 3.46 ± 0.7
h²= 0.51
P_S1
P_S2
P_R2
P_R1
Number of RILs
Adjusted means score
ADI1
n= 111
m= 2.22 ± 0.4
h²= 0.36
ADI2
n= 111
m= 2.83 ± 0.5
h²= 0.65
ADI3
n= 111
m= 3.06 ± 0.5
h²= 0.57
P_R1
P_S1
P_R2
P_S2
P_R3
P_S3
2003
LeSueur (US)
Number of RILs
Adjusted means score
