# Supplementary material for: QTL meta-analysis provides a comprehensive view of loci controlling partial resistance to Aphanomyces euteiches in four sources of resistance in pea
Source: BMC Plant Biol. 2013 Mar 16;13:45. doi: 10.1186/1471-2229-13-45 (PMC3680057; doi:10.1186/1471-2229-13-45)
Supplement: Additional file 5 — Additive-effect QTL identified from the DSP x 90–2131 RIL population for resistance to A. euteiches in infected fields over 11 environments in France and in the USA, and in controlled conditions using six pure-culture strains of A. euteiches. Values of QTL parameters were obtained using Windows QTL Cartographer 2.5 (LOD ≥ 2.9). The QTL are ordered by position on the LG. a Based on common markers, QTL common to Hamon et al. (2011) were named as they were previously. b Field scoring traits are coded as follows: location (RI = Riec/Belon (FR); DI = Dijon (FR), TPX = Templeux (FR), PLM = Pullman (USA); LS = LeSueur (USA)); year (2000 = 00; 2001 = 01; 2002 = 02; 2003 = 03); criterion (RRI = Root Rot Index; ADI = Aerial Decline Index). Controlled-condition scoring traits are indicated by the name of the strain (RB84, Ae78, Ae85, Ae87, Ae106 and Ae109). c QTL position from the first marker of the linkage group (in centimorgans Kosambi) d Nearest left marker from the LOD score peak of the QTL e Log of likelihood ratio (LOD) value at the LOD peak of the QTL for each variable f Position of the lower and upper of the one-LOD drop-off confidence interval of the QTL, from the first marker of the linkage group (in centimorgans Kosambi) g Percentage of phenotypic variance explained by an individual QTL h Effect of substituting DSP alleles for 90 2131 alleles at the QTL. A positive sign indicates that QTL alleles increasing the resistance are contributed by the resistant parent 90–2131, whereas a negative sign means that resistant alleles are brought by the susceptible parent DSP. [file 1471-2229-13-45-S5.pdf]

LG	QTL name[a]	Scoring trait[b]	Position (cM)[c]	Left marker[d]	LOD[e]	LOD-1 support interval (cM)[f]	R^2 (%)[g]	Additive effect[h]
I	*Ae-Ps1.3*	PLM03_ADI2	2.0	AA121	4.6	0.0-8.9	4.7	0.23
	Ae-Ps1.4	Ae106	41.5	AB132	4.2	33.9-45.7	7.9	-0.09
	Ae-Ps1.1	LS01_ADI	106.0	A04_430	3.3	98.5-116.6	6.6	0.13
IIa	*Ae-Ps2.1*	LS00_ADI2	42.4	AB113	6.2	37.5-44.5	14.9	-0.13
	Ae-Ps2.2	RI03_RRI	76.9	AA372.1	3.9	60.0-84.5	12.1	-0.11
IIb	*Ae-Ps2.4*	TPX03_ADI	14.0	AD85a	4.9	6.0-28.4	14.3	-0.47
		DI03_ADI1	24.4	B12_1200	4.1	21.4-28.4	9.4	-0.21
	Ae-Ps2.5	LS02_ADI1	41.9	I11_800	3.4	36.9-43.3	5.2	0.15
IIIa	*Ae-Ps3.1*	DI03_ADI2	18.1	AD57	3.0	7.2-29.5	6.0	0.21
		LS02_ADI1	20.1	AD57	5.4	16.5-29.3	9.4	0.21
		LS03_ADI3	20.1	AD57	3.2	14.9-30.4	9.1	0.15
		LS02_ADI2	38.6	O01_1900	3.3	22.9-43.0	6.8	0.18
		RI02_RRI	40.2	R04_2300	4.2	37.8-41.9	8.8	0.11
		Ae85	41.9	V03_950	3.3	40.2-46.0	7.1	0.24
IIIb	*Ae-Ps3.2*	PLM00_ADI	6.8	I11_1100	3.1	1.8-10.7	4.3	0.24
IV	*Ae-Ps4.1*	RI02_RRI	23.7	AA219	4.1	17.2-27.6	8.4	0.1
		RI03_ADI2	25.7	AA219	3.1	11.4-32.1	7.5	0.2
		RI03_ADI1	32.1	AC39c	4.3	27.0-41.9	12.9	0.18
	Ae-Ps4.2	PLM03_ADI2	79.8	N14_1900	3.2	71.9-89.1	3.1	-0.18
		PLM03_ADI1	85.8	N14_1900	3.7	79.1-91.2	3.9	-0.26
	Ae-Ps4.3	PLM00_ADI	115.5	AD134	3.7	107.3-122.7	5.0	-0.27
		DI03_RRI	122.4	AB49	5.1	121.1-126.2	14.0	0.04
		Ae78	130.2	I11_1900	5.3	126.0-134.3	9.7	0.13
		Ae87	134.9	AD249b	16.7	132.0-137.5	36.9	0.29
	Ae-Ps4.4	LS03_ADI1	157.1	AA61	4.5	151.3-167.2	21.4	-0.19
		DI03_ADI2	170.2	AA122	3.6	163.3-172.4	6.2	-0.22
		LS02_ADI1	184.1	AA378	3.3	179.0-186.6	4.9	-0.15

		DI03_ADI1	193.8	AD171	5.0	190.3-200.7	13.8	-0.26
		Ae109	204.4	O01_560	3.1	200.4-208.6	5.1	-0.08
V	*Ae-Ps5.1*	Ae85	0.0	AA81	6.4	0.0-2.0	14.3	0.22
		Ae106	3.5	LD	11.4	1.9-7.6	25.8	0.17
		Ae109	3.5	LD	15.8	2.0-8.2	37.8	0.22
		RB84	3.5	LD	6.2	0.2-8.5	7.0	0.1
		Ae78	7.5	LD	10.4	4.4-14.1	18.8	0.18
		Ae87	9.5	LD	3.5	3.5-14.9	12.7	0.17
		LS02_ADI2	16.1	R	4.1	10.9-21.0	8.3	0.2
	Ae-Ps5.4	Ae106	44.9	AD158	3.9	42.0-46.9	7.4	-0.09
	Ae-Ps5.2	DI02_ADI2	76.3	J14_1500	3.2	67.5-84.8	8.0	0.2
		DI02_ADI1	78.4	J14_1500	6.6	70.2-86.3	17.5	0.29
	Ae-Ps5.3	LS03_ADI3	121.6	P11_1200	3.6	113.9-128.5	11.8	0.17
VI	*Ae-Ps6.1*	DI03_ADI1	12.0	C14_1600	3.4	5.9-20.9	8.7	-0.2
		DI02_ADI2	13.7	AD51	3.7	6.8-21.7	6.9	-0.2
		DI02_ADI1	23.7	AD51	3.5	11.1-29.4	14.0	-0.27
	Ae-Ps6.2	LS03_ADI1	61.7	J14_750	4.8	57.2-65.8	13.7	0.19
	Ae-Ps6.3	LS03_ADI1	85.6	AD60	3.2	84.6-94.6	8.1	-0.15
	Ae-Ps6.4	RI02_RRI	117.3	E11_600	3.3	104.7-124.1	7.1	0.09
		LS00_ADI2	131.3	E11_600	4.9	124.5-140.9	14.8	0.13
		Ae78	137.2	A11_850	3.5	129.9-143.3	5.0	-0.09
VII	*Ae-Ps7.3*	DI02_ADI1	64.1	AD70	3.1	60.0-81.0	6.4	0.18
	Ae-Ps7.4	RB84	89.9	AB133	3.4	84.9-94.7	4.7	-0.1
		Ae78	91.9	AB133	3.4	83.8-94.3	5.2	-0.11
	Ae-Ps7.5	LS02_ADI1	126.3	AB36	3.5	115.7-135.6	6.3	0.18
	Ae-Ps7.6	LS03_ADI1	156.4	AA317	3.2	151.4-162.7	7.8	0.12
		DI03_ADI2	162.4	AA317	9.7	159.3-164.7	22.0	0.4
		LS03_ADI3	162.4	AA317	5.0	157.1-164.4	20.9	0.31
		PLM03_ADI1	162.4	AA317	11.0	159.3-163.8	27.2	0.89
		PLM03_ADI2	162.4	AA317	14.5	159.9-163.5	38.3	0.96
		TPX03_ADI	162.5	AA317	4.5	157.4-179	13.4	0.46
		DI03_ADI1	164.4	AA317	6.2	159.4-174.8	16.1	0.27

LS01_ADI	167.5	A11_1200	6.6	167.3-169.5	19.1	0.2
Ae85	171.9	PSARGDEC	5.9	165.5-174.9	13.5	0.21
RI03A_ADI1	171.9	PSARGDEC	2.9	166.3-176.6	8.5	0.17
LS00_ADI2	172.8	AB27	13.7	172.4-173.9	37.9	0.21
LS02_ADI1	172.8	AB27	11.8	172.4-174.8	20.9	0.32
LS02_ADI2	172.8	AB27	7.4	172.2-178.0	16.1	0.33
PLM00_ADI	172.8	AB27	18.0	172.6-174.1	34.5	0.77
RI02_RRI	172.8	AB27	10.7	172.2-174.0	25.6	0.17
RI03_RRI	172.8	AB27	5.9	172.5-179.1	12.2	0.13
DI02_ADI2	174.8	AB27	4.3	172.2-179.4	8.4	0.22
PLM03_ADI1	174.8	AB27	10.2	172.8-175.6	13.4	0.64
PLM03_ADI2	174.8	AB27	15.6	172.8-175.6	21.6	0.64
RI03_ADI2	174.8	AB27	7.6	171.4-177.1	19.9	0.27
DI02_ADI1	175.0	AA224a	7.2	174.0-177.6	15.1	0.29
LS00_ADI1	175.0	AA224a	7.3	174.9-177.6	29.5	0.2
RB84	177.0	AA224a	27.4	176.3-179.3	59.8	0.3
Ae106	179.5	AB122b	11.2	176.9-180.4	24.7	0.15
Ae109	179.5	AB122b	7.7	177.7-181.9	13.4	0.13
Ae78	179.5	AB122b	12.8	177.8-181.1	22.9	0.2
DI03_RRI	184.4	AB30	3.1	181.8-186.3	7.4	0.03
