# Supplementary material for: QTL meta-analysis provides a comprehensive view of loci controlling partial resistance to Aphanomyces euteiches in four sources of resistance in pea
Source: BMC Plant Biol. 2013 Mar 16;13:45. doi: 10.1186/1471-2229-13-45 (PMC3680057; doi:10.1186/1471-2229-13-45)
Supplement: Additional file 8 — Additive-effect QTL identified from the DSP x 90–2131, Baccara x PI180693 and Baccara x 552 RIL populations for flowering time and plant height traits, scored in eight infested or healthy environments (values obtained by Windows QTL Cartographer 2.5, LOD ≥ 2.9 for the DSP x 90-2131 RIL population and LOD ≥ 2.8 for the Baccara x PI180693 and Baccara x 552 RIL populations). The QTL are ordered by position on the LG. a Scoring traits are coded as follows: population (D2131 = DSP x 90 2131; B552 = Baccara x 552; BPI = Baccara x PI180693); location (DI = Dijon (FR); REN = Rennes (FR)); year (02 = 2002; 03 = 2003; 04 = 2004; 05 = 2005; 06 = 2006; 01 = 2007; 08 = 2008); criterion (Flo1 and Flo2 = Flowering times; HT = Plant Height) b QTL position from the first marker of the linkage group (in centimorgans Kosambi) c Nearest left marker from the LOD score peak of the QTL d Log of likelihood ratio (LOD) value at the LOD peak of the QTL for each variable e Position of the lower and upper of the one-LOD drop-off confidence interval of the QTL, from the first marker of the linkage group (in centimorgans Kosambi) f Percentage of phenotypic variance explained by each individual QTL g Effect of substituting Baccara or DSP alleles for 552 or PI180693 or 90-2131 alleles at the QTL. A positive sign indicates that QTL alleles increasing the earliness at flowering or decreasing plant height are contributed by the resistant parent 552, or PI180693 or 90-2131, whereas a negative sign means that earliness or short plant alleles are brought by the susceptible parent Baccara or DSP. [file 1471-2229-13-45-S8.pdf]

LG	QTL name	Scoring trait[a]	Position (cM)[b]	Left marker[c]	LOD[d]	LOD-1 support interval (cM)[e]	R^2 (%)[f]	Additive effect[g]
**FLOWERING TIME**								
I	*Flo-Ps1.1*	BPI_REN08_Flo2	29.7	O09_800	3.2	19.9-33.3	3.4	-1.13
		B552_DI04_Flo1	47.8	AA474	3.8	33.2-54.7	4.8	-0.32
	Flo-Ps1.2	BPI_DI07_Flo1	107.6	D21	2.8	100.6-114.5	2.4	1.1
		B552_DI04_Flo1	111.5	Af	8.2	107.9-116.6	10	0.47
		BPI_DI06_Flo1	113.5	Af	3.3	102.7-131.3	2.3	1.17
		BPI_REN08_Flo2	113.6	Af	3.6	103.6-128.1	4.1	1.22
II	*Flo-Ps2.1*	D2131_DI03_Flo1	62.5	AB101b	3.0	58.5-66.5	10	0.86
	Flo-Ps2.2	BPI_DI06_Flo1	84.3	AB112	34	81.6-85.6	31.2	-4.42
		BPI_DI08_Flo1	84.3	AB112	22.9	81.3-86.2	23.9	-4.36
		BPI_REN08_Flo1	84.3	AB112	27.8	82.0-85.3	28.1	-4.76
		BPI_REN05_Flo1	86.7	AB50	42.6	86.3-86.8	40.8	-5.85
		BPI_DI07_Flo1	86.7	AB50	33.3	86.3-88.2	31.9	-4.16
	Flo-Ps2.3	B552_DI04_Flo1	193.2	AB40	3.3	180.9-202.8	4.2	0.32
III	*Flo-Ps3.1*	BPI_REN08_Flo2	41.4	X03_1000	23.8	39.2-44.0	32.6	-3.38
		BPI_DI08_Flo1	43.4	X03_1000	21.6	40.2-44.6	22.6	-4.11
		BPI_REN08_Flo1	43.4	X03_1000	16.5	40.7-44.0	14.7	-3.05
		BPI_DI07_Flo1	45.0	X03_1000	17.9	41.6-47.5	14.1	-2.81
		BPI_REN05_Flo1	43.4	X03_1000	22.8	41.1-49.7	18.7	-3.92
		BPI_DI06_Flo1	47.0	AB28b	21	42.5-48.7	17.2	-3.21
IV	*Flo-Ps4.1*	D2131_REN02_Flo1	170.0	AA122	6.2	157.8-176.8	11	0.67
	Flo-Ps4.2	B552_DI04_Flo1	236.4	AD171	6.1	232.3-242.2	8.1	0.42
V	*Flo-Ps5.1*	B552_DI04_Flo1	170.3	AA460	3	163.1-183.1	3.5	0.27
VI	*Flo-Ps6.1*	B552_DI04_Flo1	27.8	PSBT2AGEN	5.2	26.0-29.0	6.2	-0.39
	Flo-Ps6.2	B552_DI04_Flo1	94.6	AA224	10.9	91.3-97.4	16.3	-0.59
	Flo-Ps6.3	D2131_DI03_Flo1	72.0	O01_700	5.2	70.3-74.3	13.9	0.93
		D2131_REN02_Flo1	69.0	AA31	13.9	69.1-70.5-	40	1.34
VII	*Flo-Ps7.1*	D2131_REN02_Flo1	0.0	E11_900	4.5	0.0-5.6	11	-0.67
	Flo-Ps7.2	BPI_REN05_Flo1	252.3	B12_850	25.1	252.2-260.0	26.6	5.03
		BPI_DI06_Flo1	260.8	AA176	38.8	259.4-261.8	36.4	5.11
		BPI_DI08_Flo1	260.8	AA176	35.6	260.0-263.3	37.5	5.47
		BPI_REN08_Flo2	260.8	AA176	29.5	260.0-264.6	40.7	3.9
		BPI_DI07_Flo1	262.8	AA176	37	260.8-264.0	38.4	4.68
		BPI_REN08_Flo1	262.8	AA176	33.1	260.3-263.8	34.4	4.99
**PLANT HEIGHT**								
III	*HT-Ps3.1*	D2131_REN02_HT	155.6	AB68	3.3	151.8-159.8	10.6	5.4
IV	*HT-Ps4.1*	D2131_REN02_HT	228.1	X18_600	4.6	195.7-246.6	10.0	4.0
V	*HT-Ps5.1*	D2131_REN02_HT	96.6	A19_960	4.9	90.3-104.6	16.9	5.0
