# Supplementary material for: QTL meta-analysis provides a comprehensive view of loci controlling partial resistance to Aphanomyces euteiches in four sources of resistance in pea
Source: BMC Plant Biol. 2013 Mar 16;13:45. doi: 10.1186/1471-2229-13-45 (PMC3680057; doi:10.1186/1471-2229-13-45)
Supplement: Additional file 9 — Localization of individual QTL and meta-QTL for resistance and morphological traits onto the consensus marker map: linkage group VII (for legend, see Figure 2). [file 1471-2229-13-45-S9.pdf]

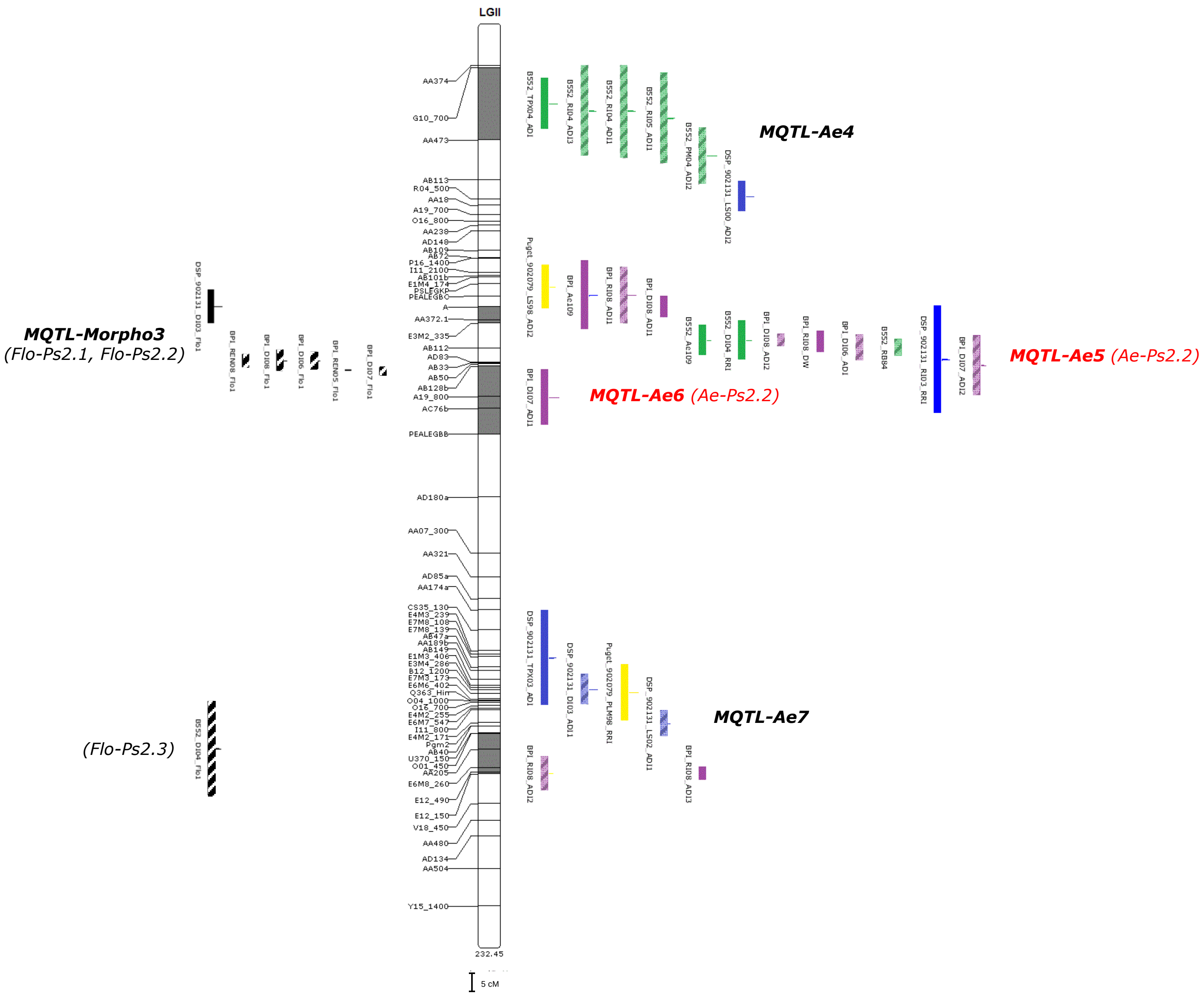

LGII
MQTL-Ae4
MQTL-Morpho3
(Flo-Ps2.1, Flo-Ps2.2)
MQTL-Ae6 (Ae-Ps2.2)
MQTL-Ae5 (Ae-Ps2.2)
(Flo-Ps2.3)
MQTL-Ae7
AA374
G10_700
AA473
AB113
R04_500
AA18
A19_700
O16_800
AA238
AD148
AB109
AB72
P16_1400
I11_2100
AB101b
E1M4_174
PSLEGKP
PEALEGBC
A
AA372.1
E3M2_335
AB112
AD83
AB33
AB50
AB128b
A19_800
AC76b
PEALEGBB
AD180a
AA07_300
AA321
AD85a
AA174a
CS35_130
E4M3_239
E7M8_108
E7M8_139
AB47a
AA189b
AB149
E1M3_406
E3M4_286
B12_1200
E7M3_173
E6M6_402
Q363_Hin
O04_1000
O16_700
E4M2_255
E6M7_547
I11_800
E4M2_171
Pgm2
AB40
U370_150
O01_450
AA205
E6M8_260
E12_490
E12_150
V18_450
AA480
AD134
AA504
Y15_1400
232.45
5 cM

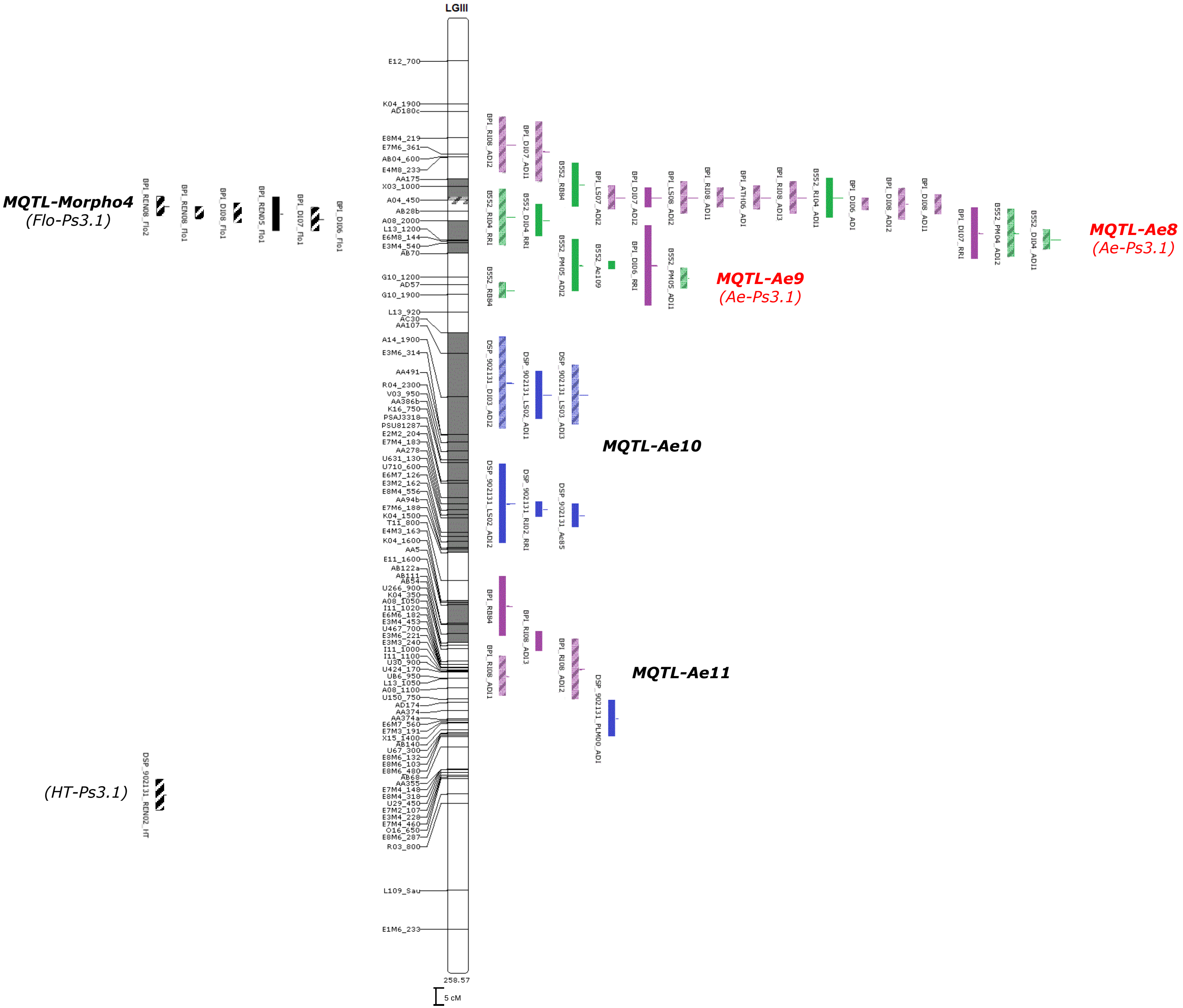
LGIII
MQTL-Morpho4
(Flo-Ps3.1)
(HT-Ps3.1)
MQTL-Ae8
(Ae-Ps3.1)
MQTL-Ae9
(Ae-Ps3.1)
MQTL-Ae10
MQTL-Ae11
E12_700
K04_1900
AD180c
E8M4_219
E7M6_361
AB04_600
E4M8_233
AA175
X03_1000
A04_450
AB28b
A08_2000
L13_1200
E6M8_144
E3M4_540
AB70
G10_1200
AD57
G10_1900
L13_920
AC30
AA107
A14_1900
E3M6_314
AA491
R04_2300
V03_950
AA386b
K16_750
PSAJ3318
PSU81287
E2M2_204
E7M4_183
AA278
U631_130
U710_600
E6M7_126
E3M2_162
E8M4_556
AA94b
E7M6_188
K04_1500
T11_800
E4M3_163
K04_1600
AA5
E11_1600
AB122a
AB111
AB54
U266_900
K04_350
A08_1050
I11_1020
E6M6_182
E3M4_453
U467_700
E3M6_221
E3M3_240
I11_1000
I11_1100
U30_900
U424_170
UB6_950
L13_1050
A08_1100
U150_750
AD174
AA374
AA374a
E6M7_560
E7M3_191
X15_1400
AB140
U67_300
E8M6_132
E8M6_103
E8M6_480
AB68
AA355
E7M4_148
E8M4_318
U29_450
E7M2_107
E3M4_228
E7M4_460
O16_650
E8M6_287
R03_800
L109_Sau
E1M6_233
258.57
5 cM

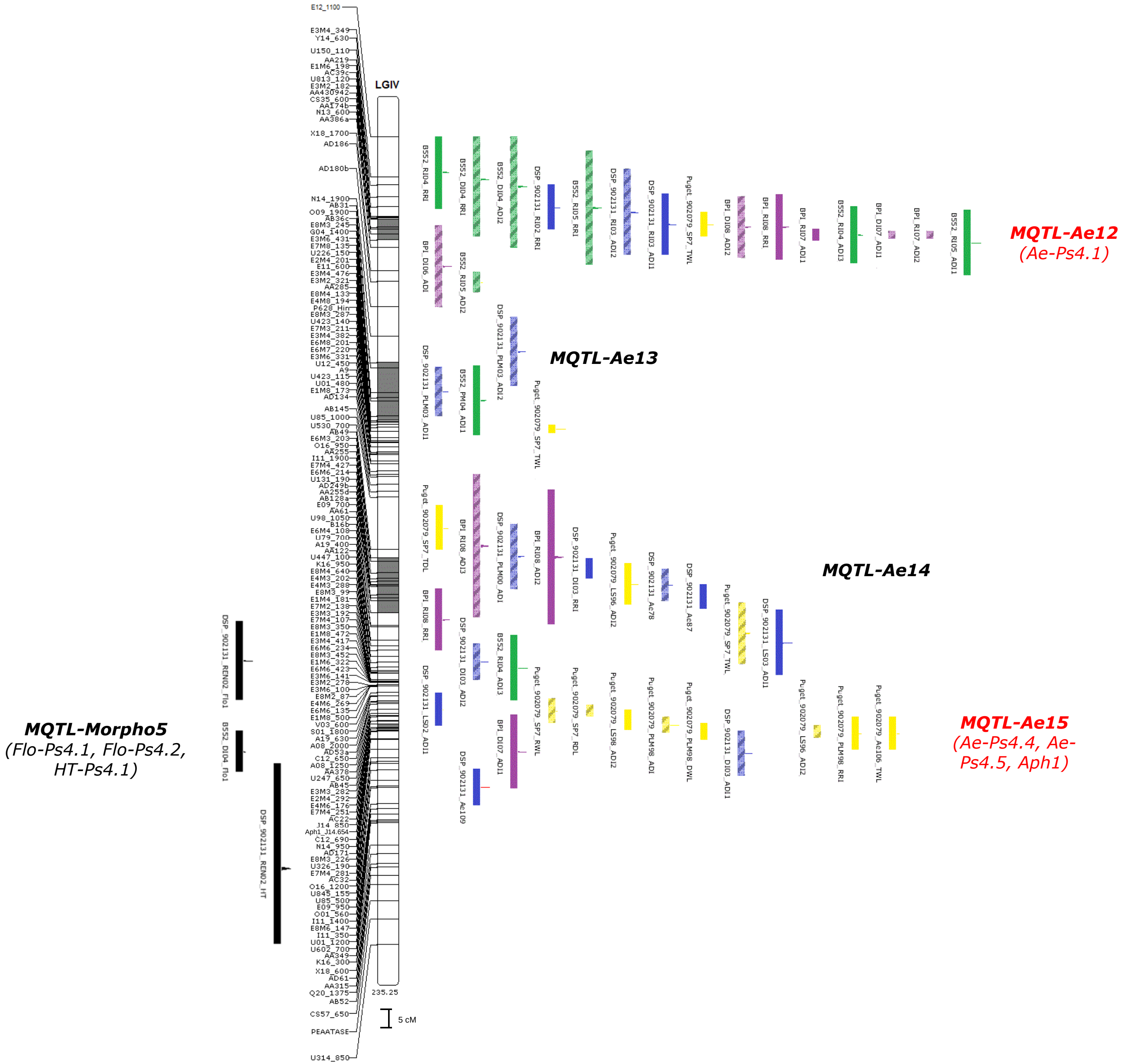
LGIV
235.25
5 cM
MQTL-Ae12
(Ae-Ps4.1)
MQTL-Ae13
MQTL-Ae14
MQTL-Ae15
(Ae-Ps4.4, Ae-Ps4.5, Aph1)
MQTL-Morpho5
(Flo-Ps4.1, Flo-Ps4.2, HT-Ps4.1)

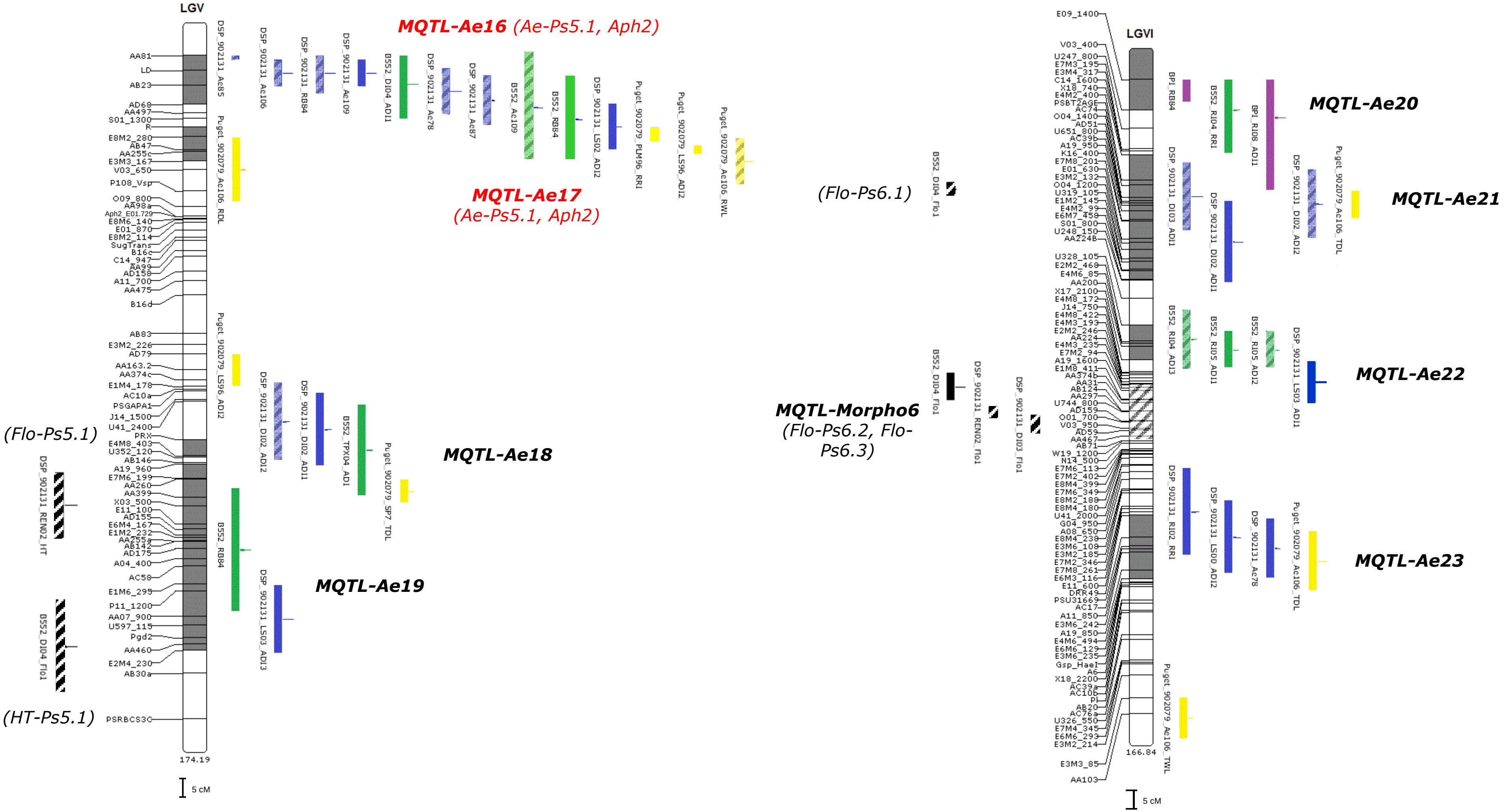
LGV
MQTL-Ae16 (Ae-Ps5.1, Aph2)
MQTL-Ae17
(Ae-Ps5.1, Aph2)
MQTL-Ae18
MQTL-Ae19
(Flo-Ps5.1)
(HT-Ps5.1)
174.19
5 cM
(Flo-Ps6.1)
MQTL-Morpho6
(Flo-Ps6.2, Flo-Ps6.3)
LGVI
MQTL-Ae20
MQTL-Ae21
MQTL-Ae22
MQTL-Ae23
166.84
5 cM

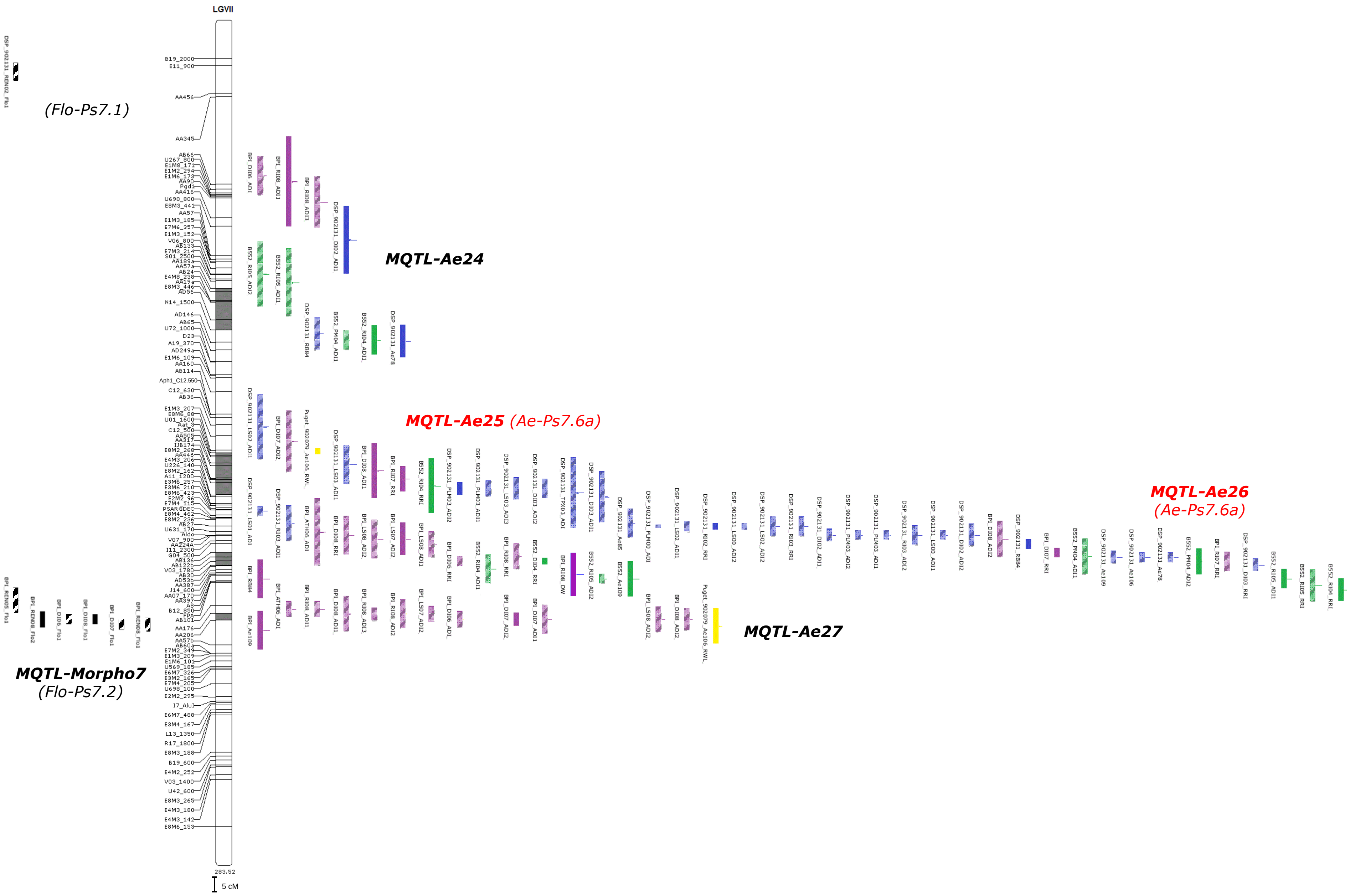
LGVII
(Flo-Ps7.1)
MQTL-Ae24
MQTL-Ae25 (Ae-Ps7.6a)
MQTL-Ae26
(Ae-Ps7.6a)
MQTL-Ae27
MQTL-Morpho7
(Flo-Ps7.2)
283.52
5 cM
